# Supplementary figures and images for: Antiviral function and viral antagonism of the rapidly evolving dynein activating adaptor NINL
Source: eLife. 2022 Oct 12;11:e81606. doi: 10.7554/eLife.81606 (PMC9651953; doi:10.7554/eLife.81606)

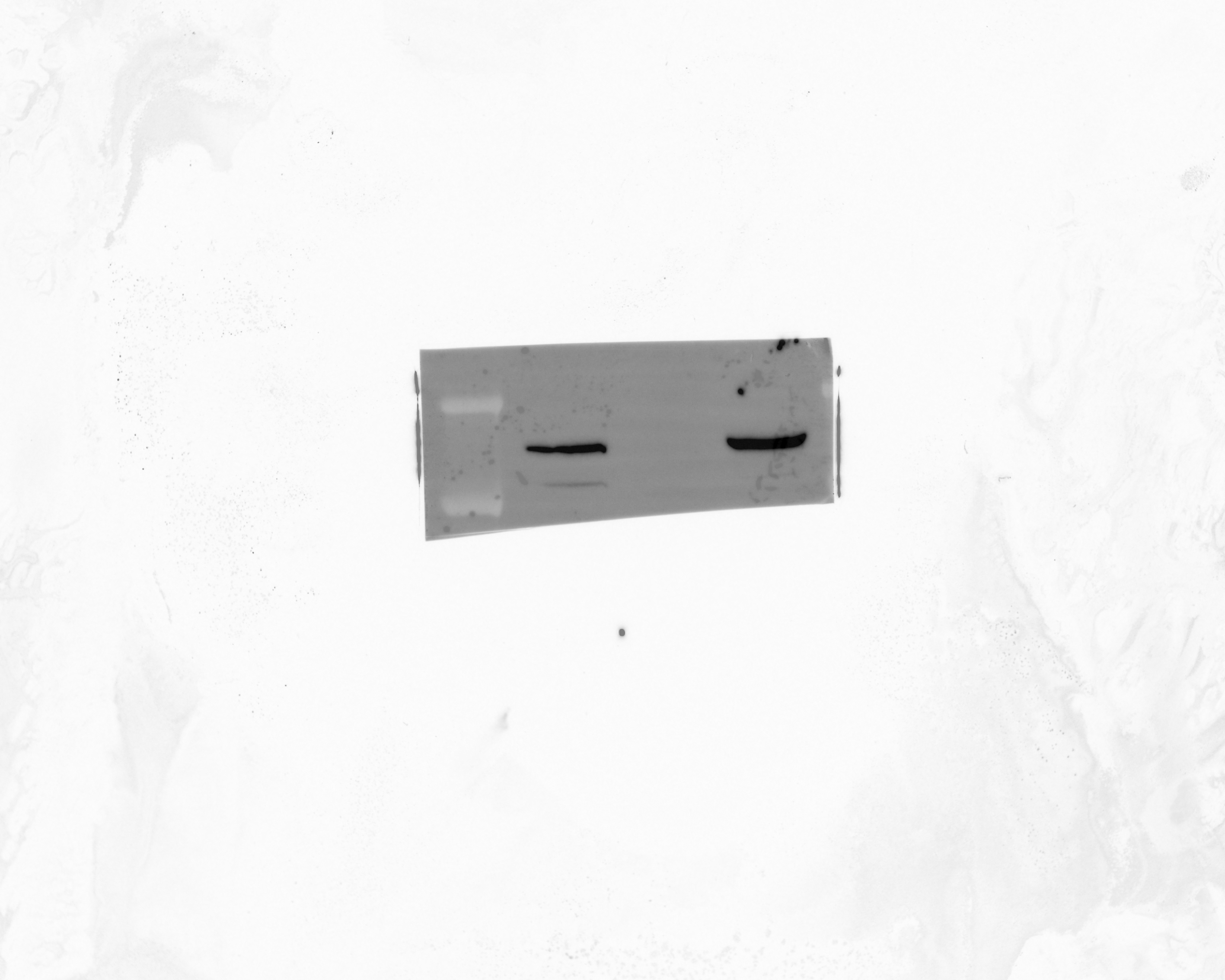

Supplement: Figure 2—source data 1. [file elife-81606-fig2-data1.zip › Figure 2/KO_Confirmation_NINL (Multichannel).tif]

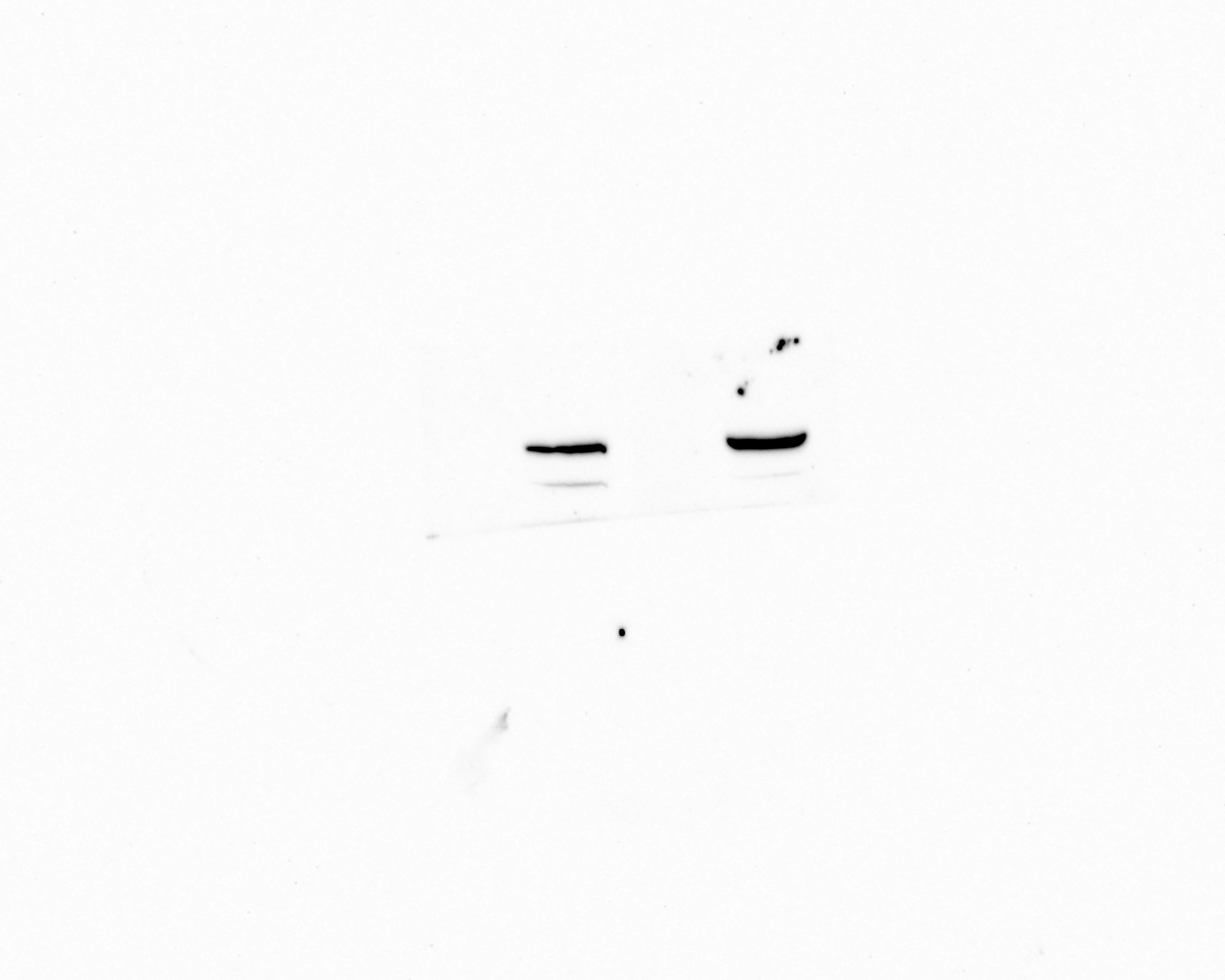

Supplement: Figure 2—source data 1. [file elife-81606-fig2-data1.zip › Figure 2/KO_Confirmation_NINL.tif]

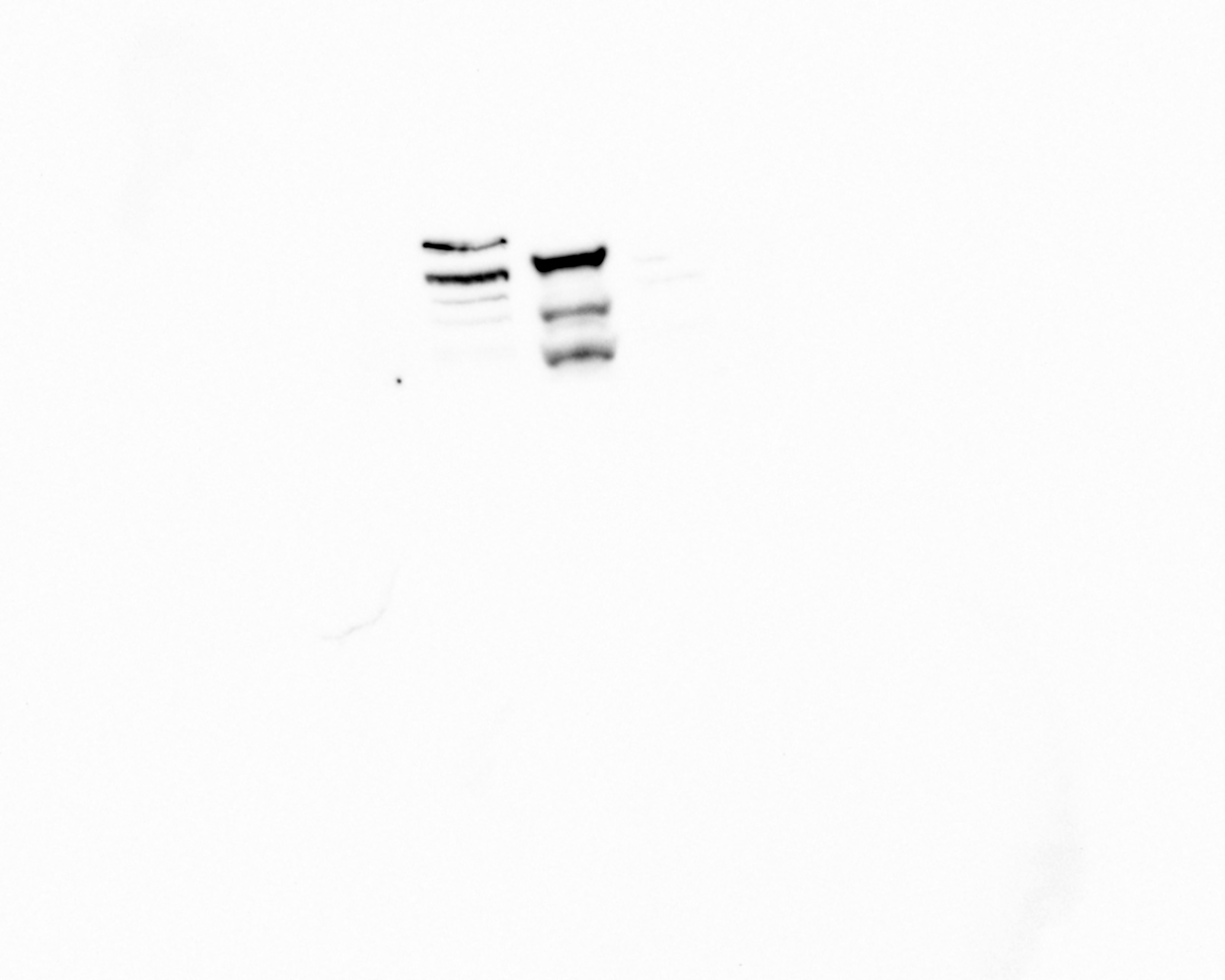

Supplement: Figure 2—source data 1. [file elife-81606-fig2-data1.zip › Figure 2/KO_Confirmation_NIN.tif]

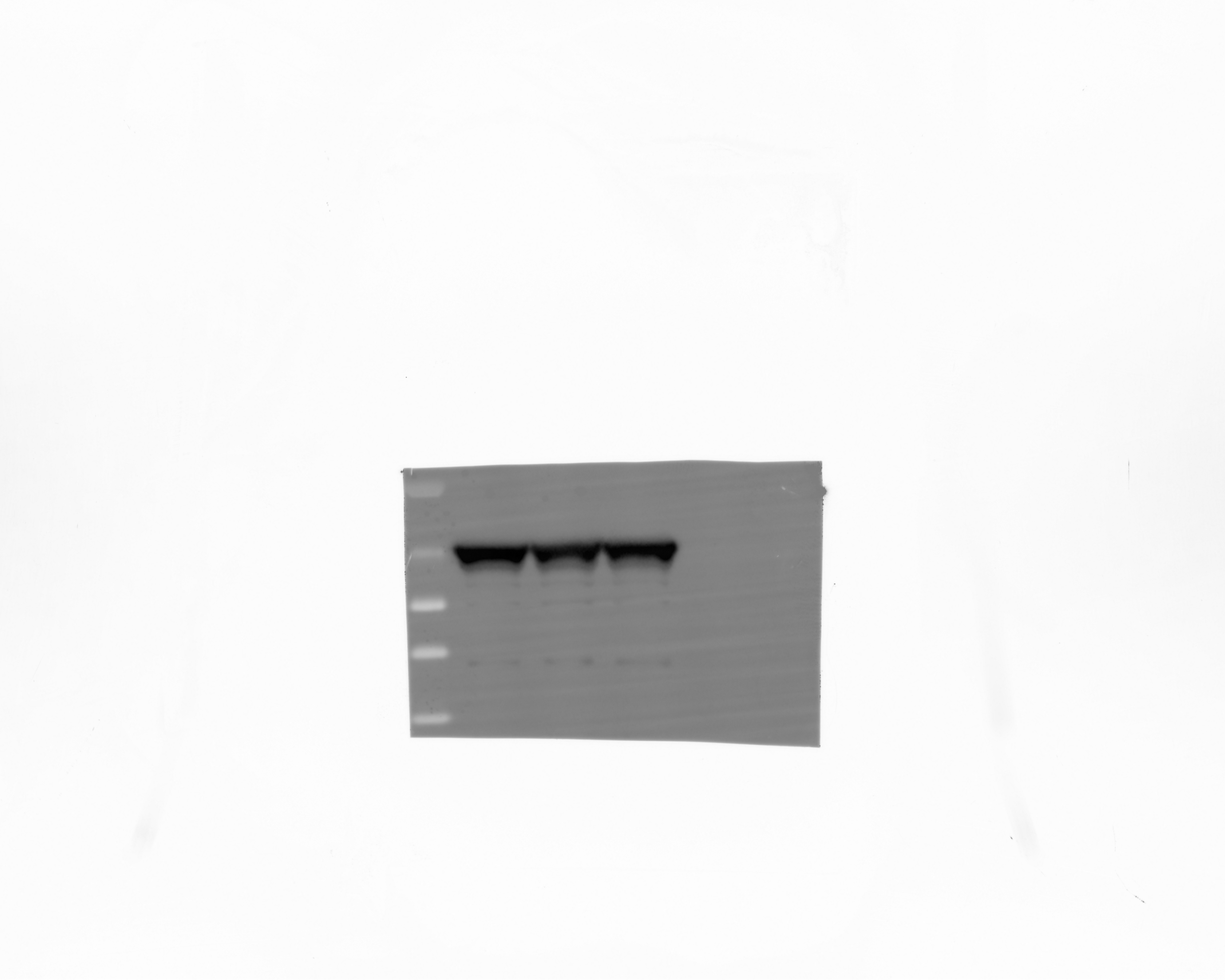

Supplement: Figure 2—source data 1. [file elife-81606-fig2-data1.zip › Figure 2/KO_Confirmation_GAPDH (Multichannel).tif]

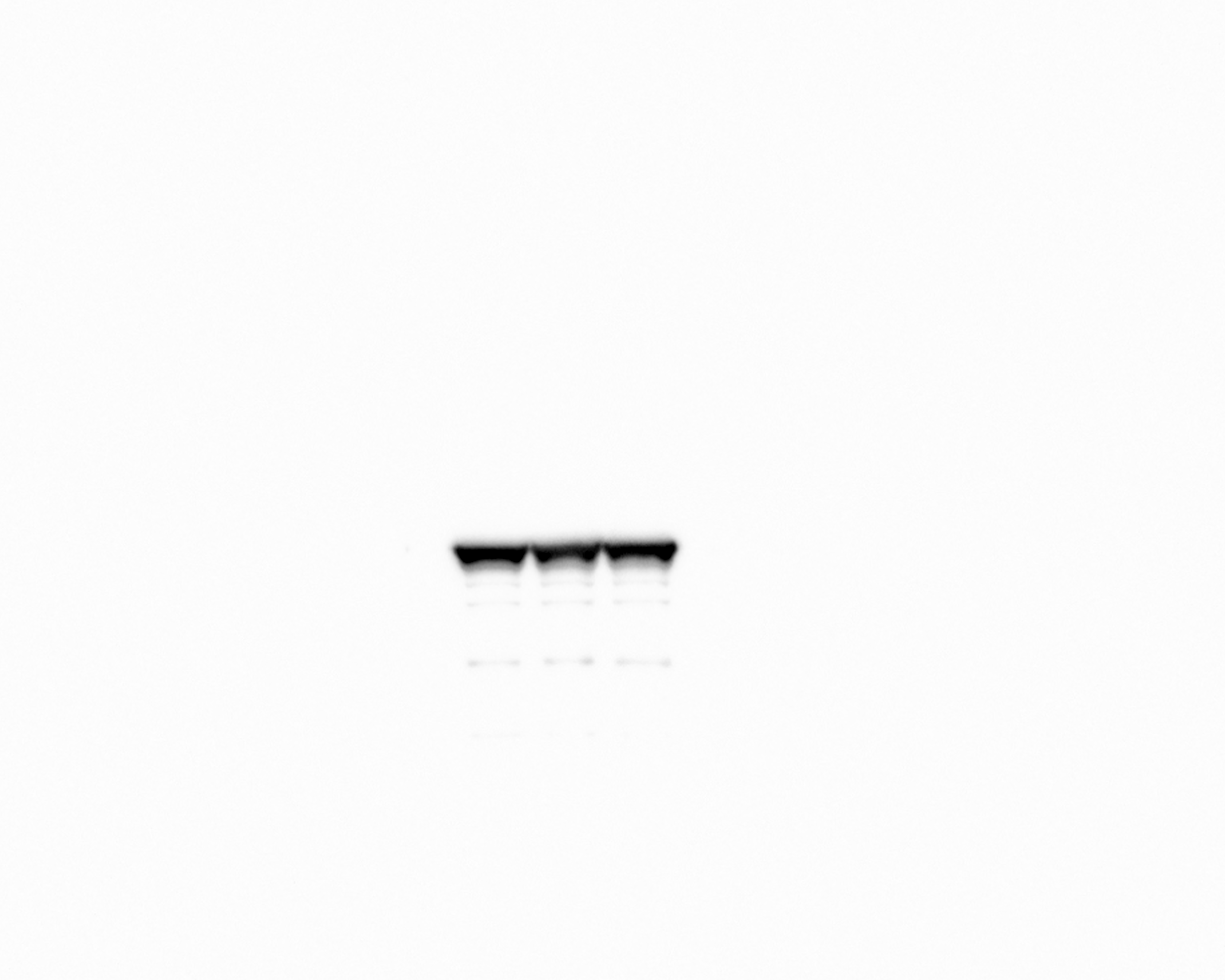

Supplement: Figure 2—source data 1. [file elife-81606-fig2-data1.zip › Figure 2/KO_Confirmation_GAPDH.tif]

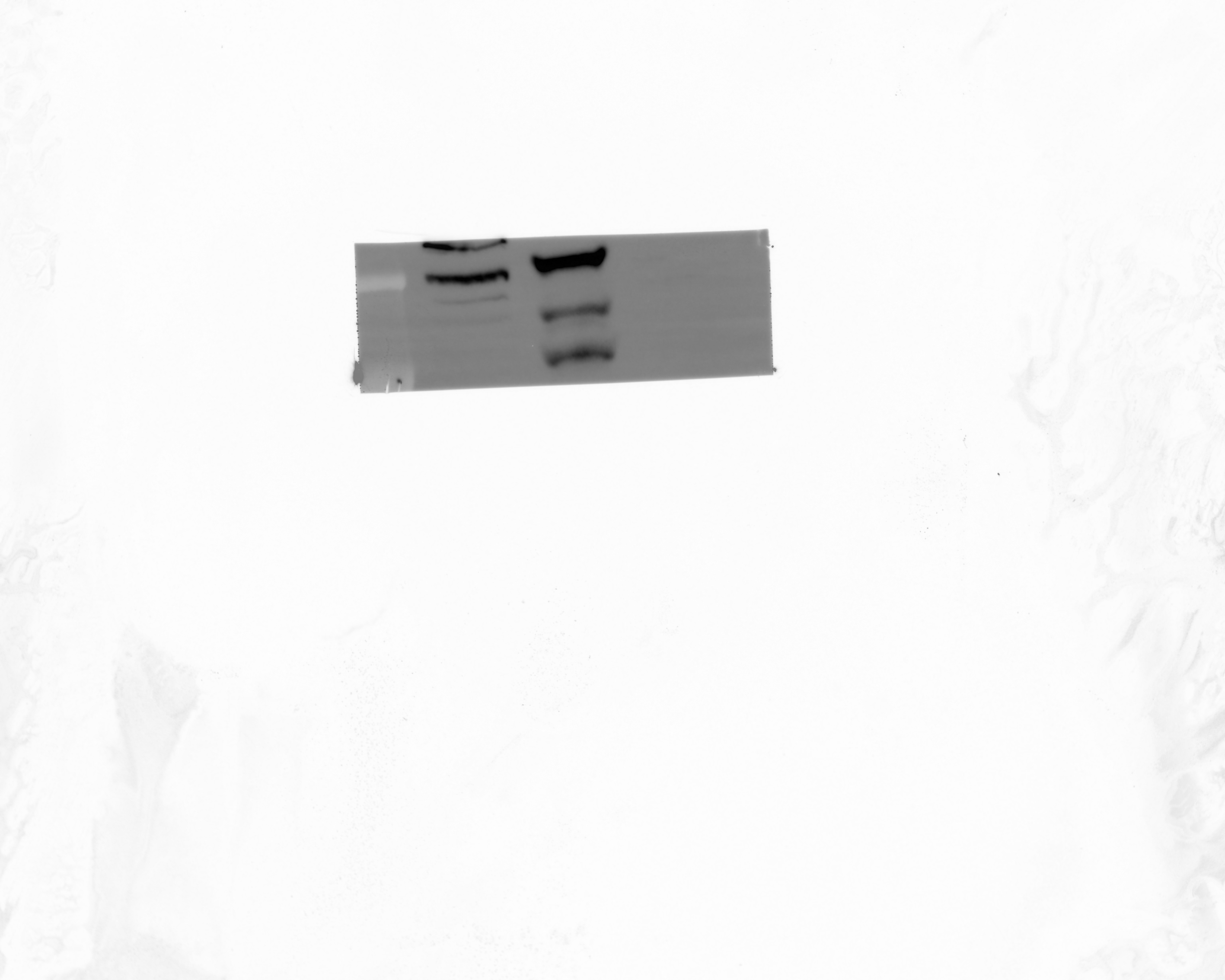

Supplement: Figure 2—source data 1. [file elife-81606-fig2-data1.zip › Figure 2/KO_Confirmation_NIN (Multichannel).tif]

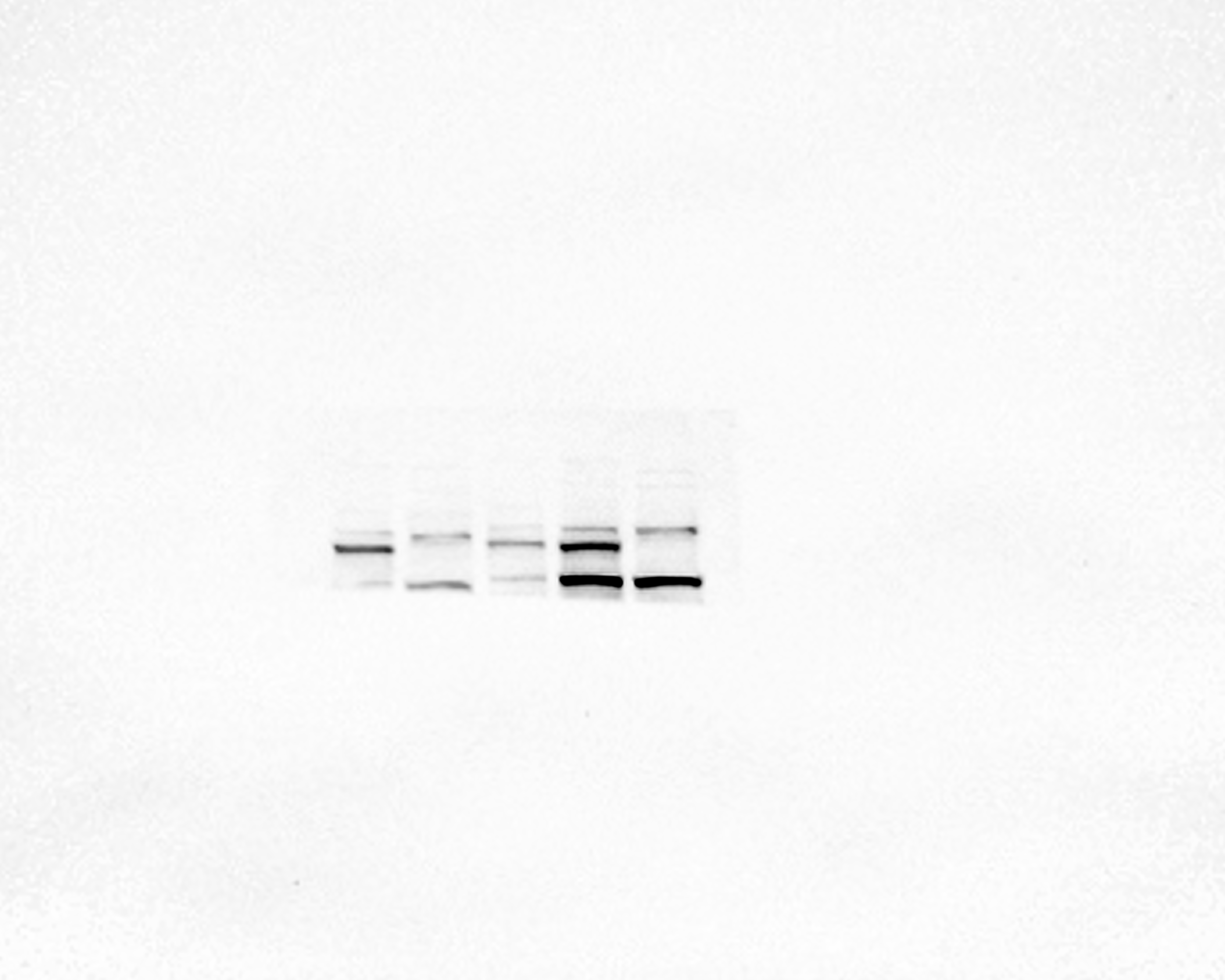

Supplement: Figure 2—figure supplement 1—source data 1. [file elife-81606-fig2-figsupp1-data1.zip › Figure 2 Figure supplement 1/KO Confirmation_hct116:u2os_ninl.tif]

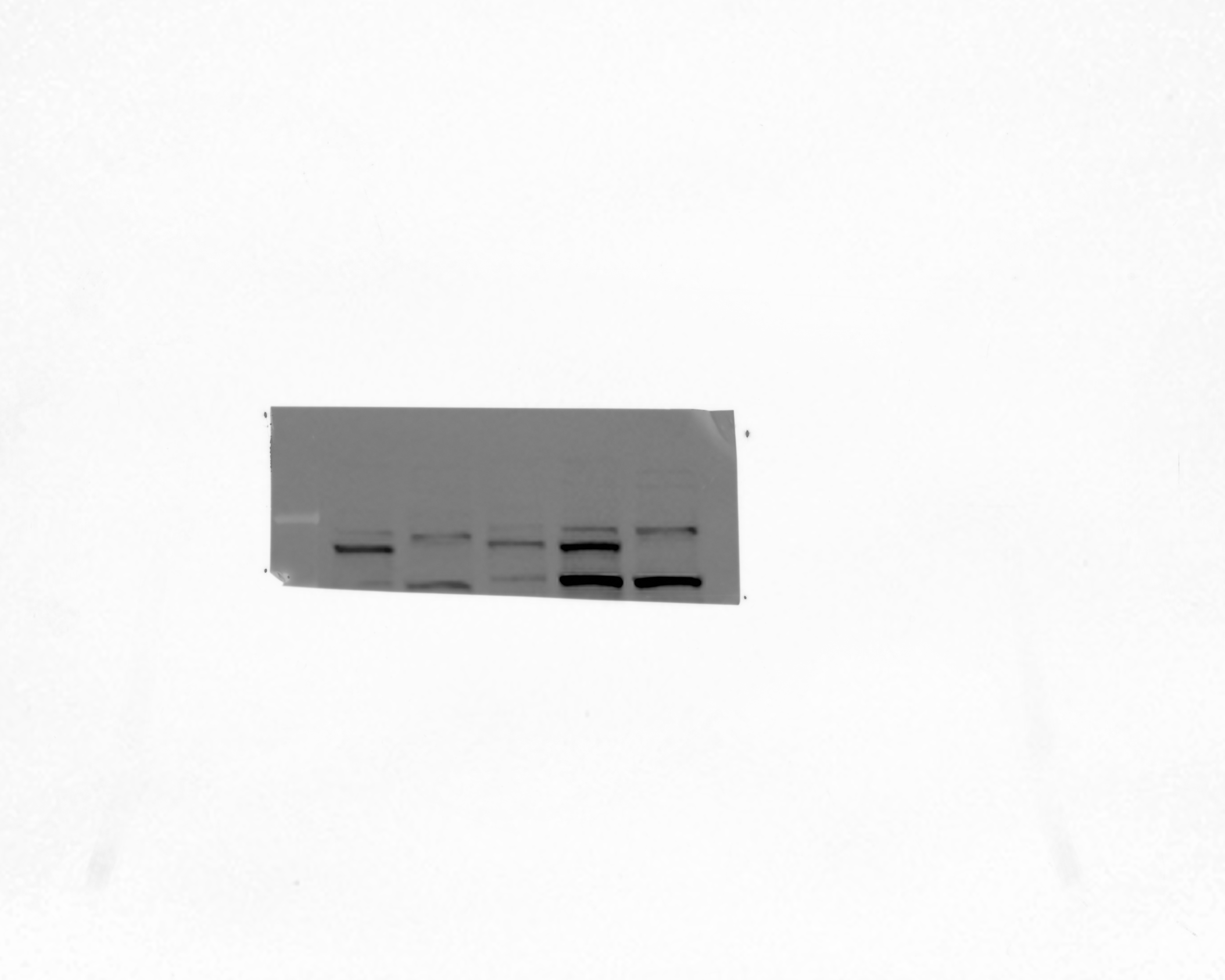

Supplement: Figure 2—figure supplement 1—source data 1. [file elife-81606-fig2-figsupp1-data1.zip › Figure 2 Figure supplement 1/KO Confirmation_hct116:u2os_ninl (Multichannel).tif]

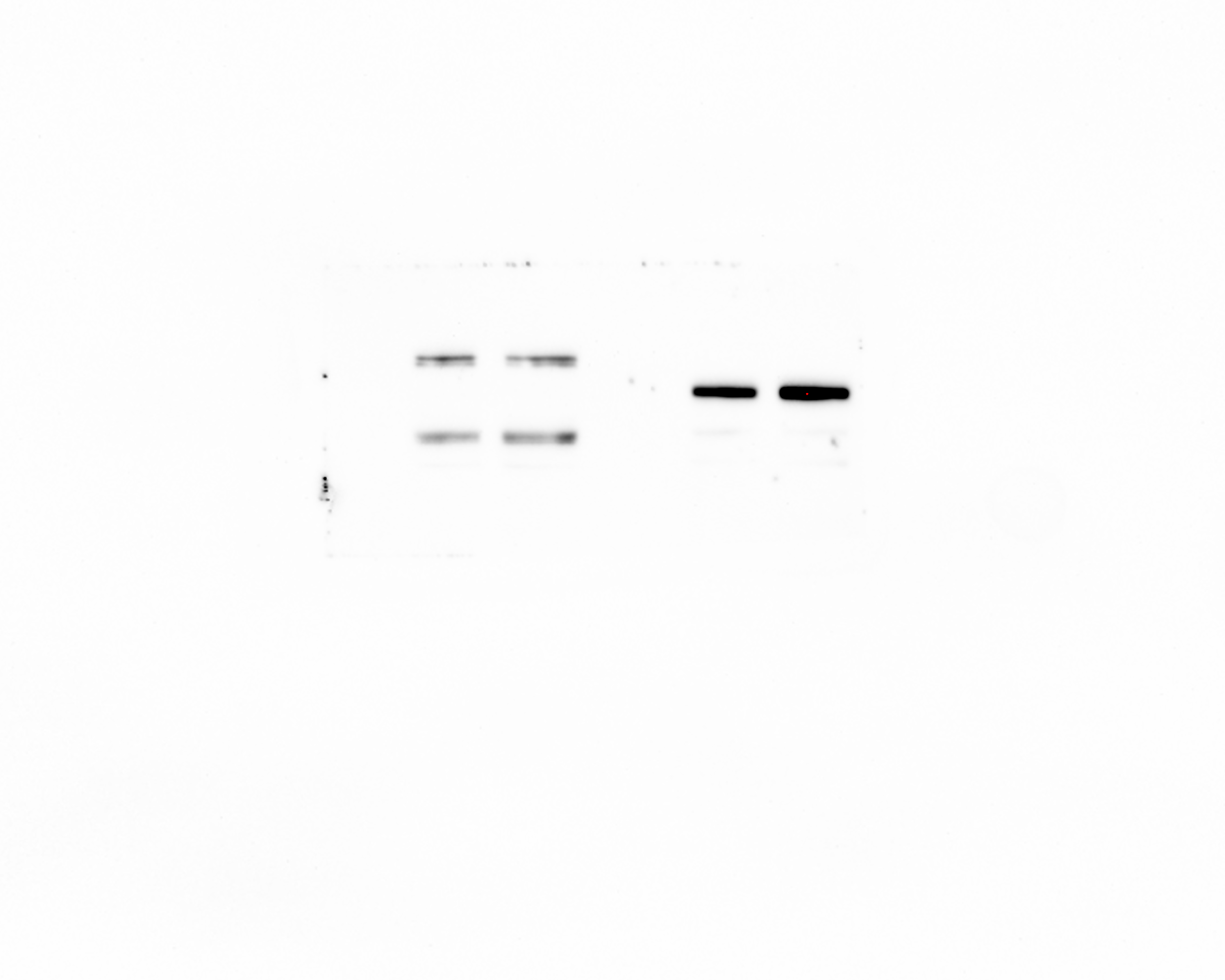

Supplement: Figure 2—figure supplement 1—source data 1. [file elife-81606-fig2-figsupp1-data1.zip › Figure 2 Figure supplement 1/KO Confirmation_hct116:u2os_nin.tif]

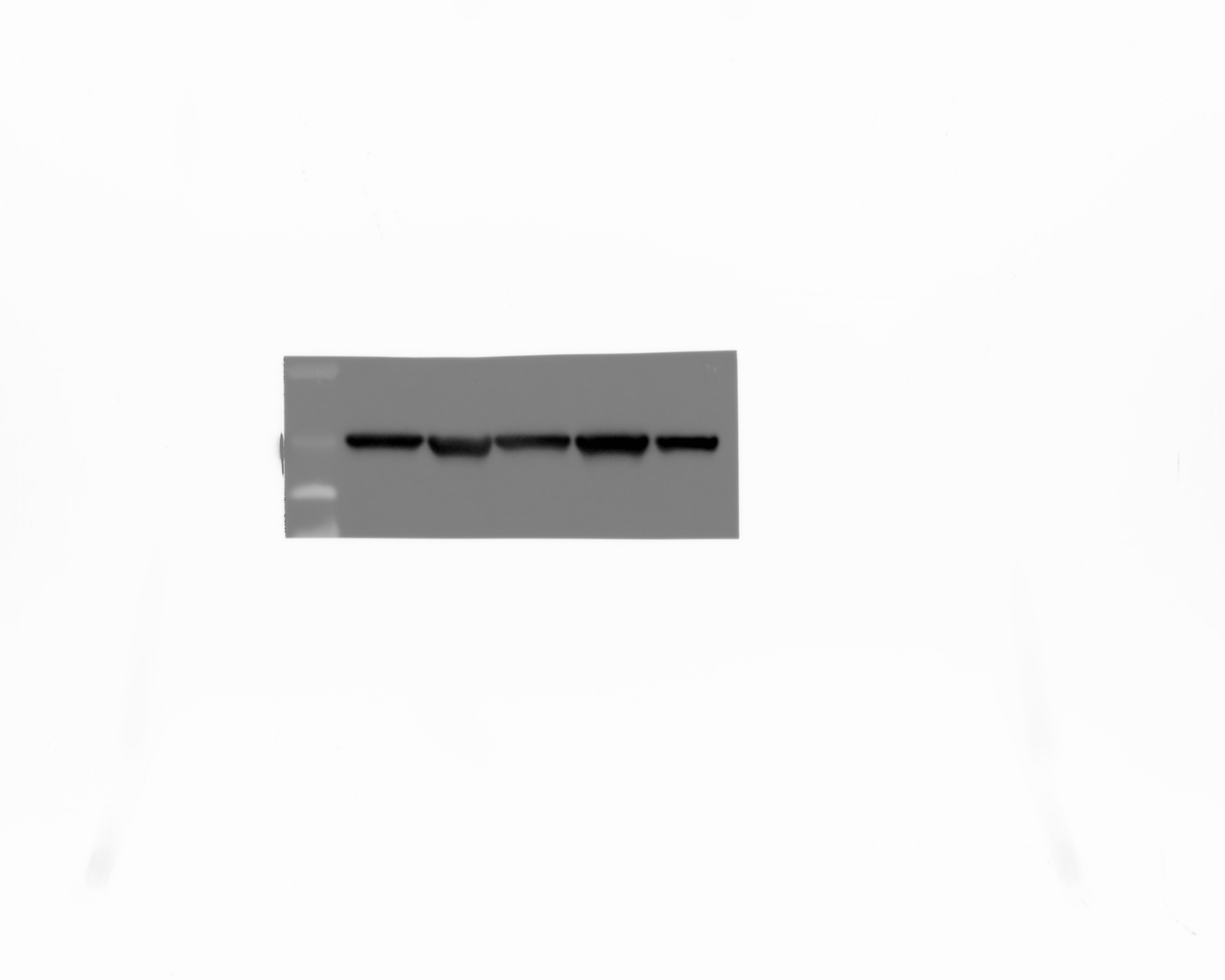

Supplement: Figure 2—figure supplement 1—source data 1. [file elife-81606-fig2-figsupp1-data1.zip › Figure 2 Figure supplement 1/KO Confirmation_hct116:u2os_gapdh (Multichannel).tif]

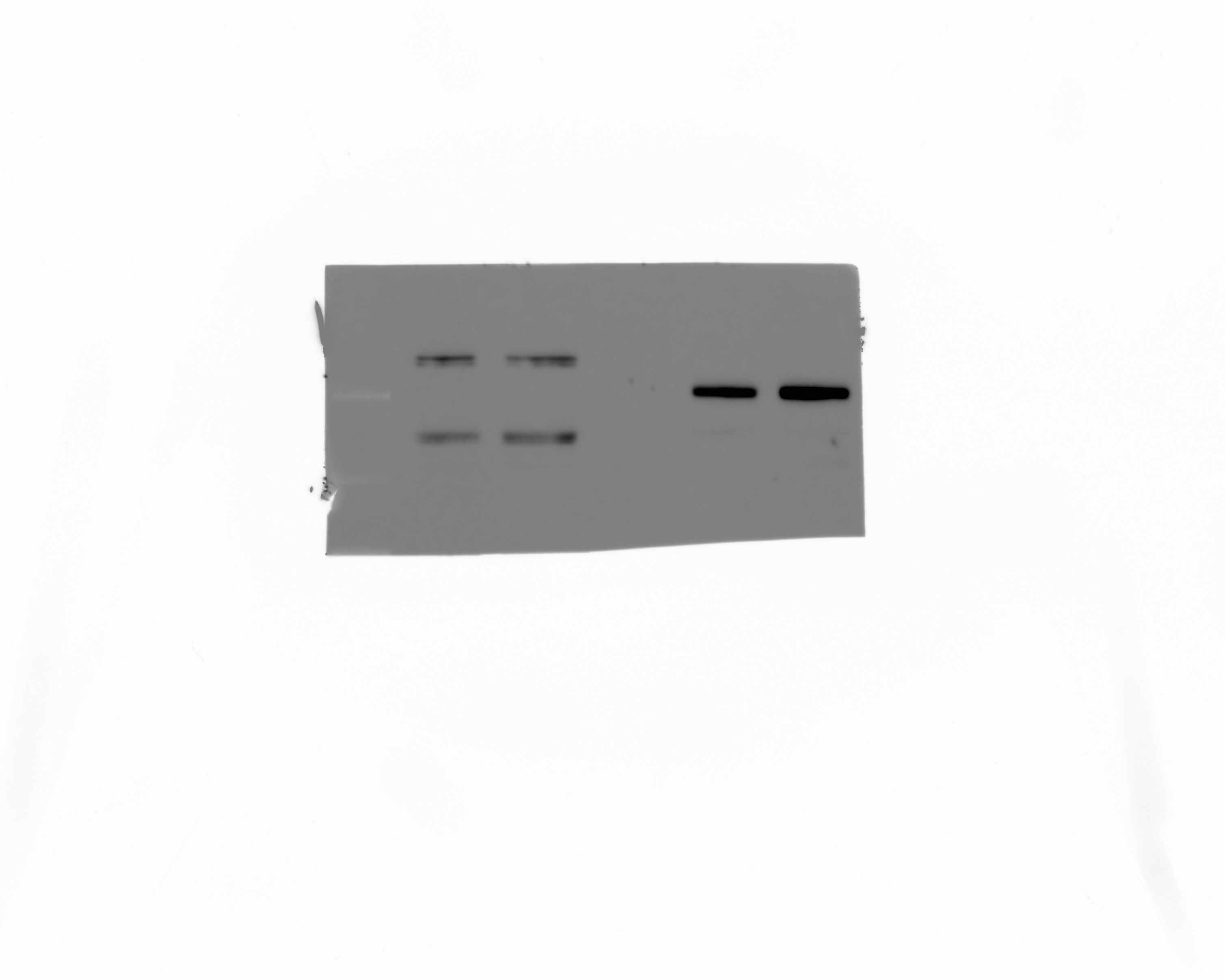

Supplement: Figure 2—figure supplement 1—source data 1. [file elife-81606-fig2-figsupp1-data1.zip › Figure 2 Figure supplement 1/KO Confirmation_hct116:u2os_nin (Multichannel).tif]

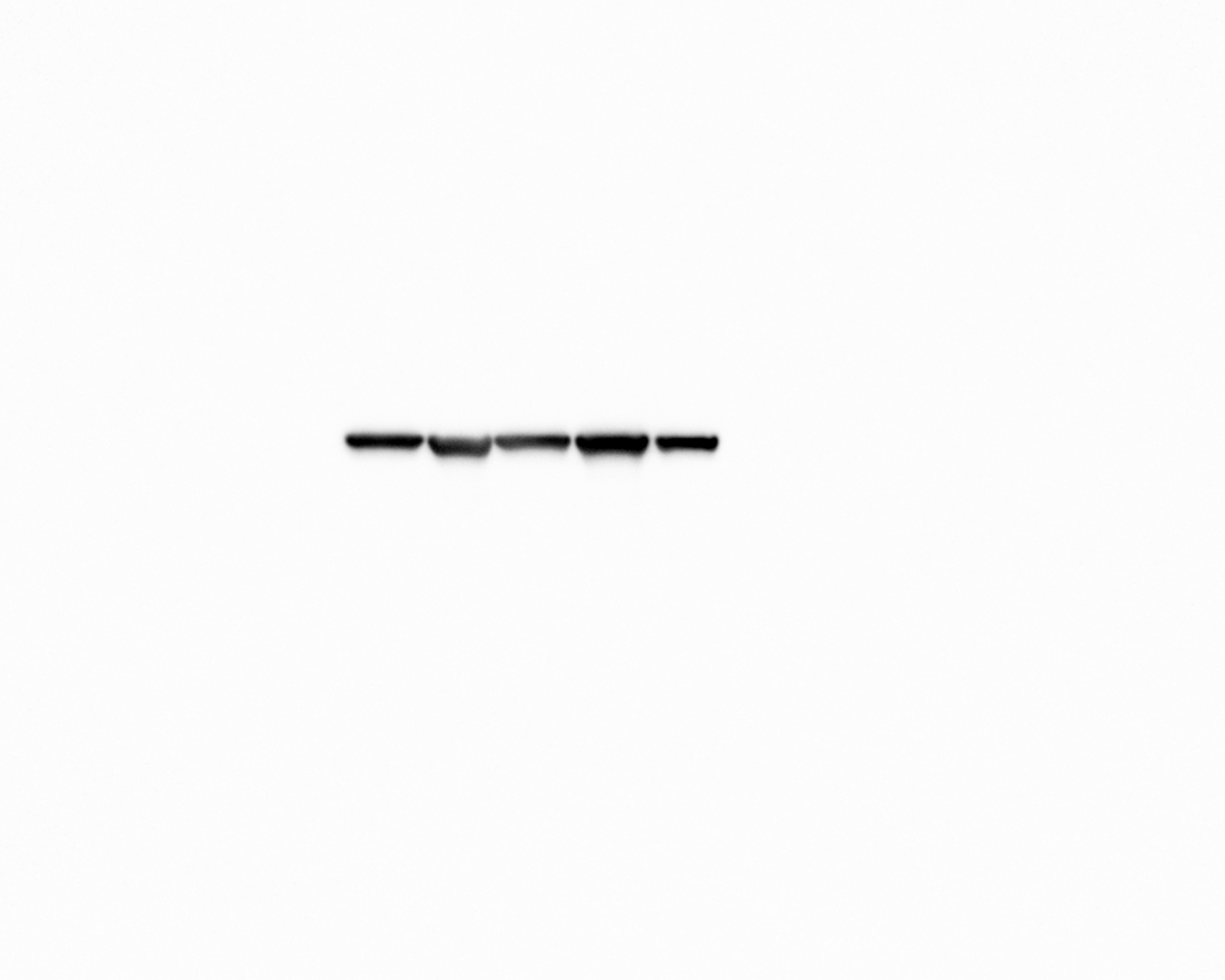

Supplement: Figure 2—figure supplement 1—source data 1. [file elife-81606-fig2-figsupp1-data1.zip › Figure 2 Figure supplement 1/KO Confirmation_hct116:u2os_gapdh.tif]

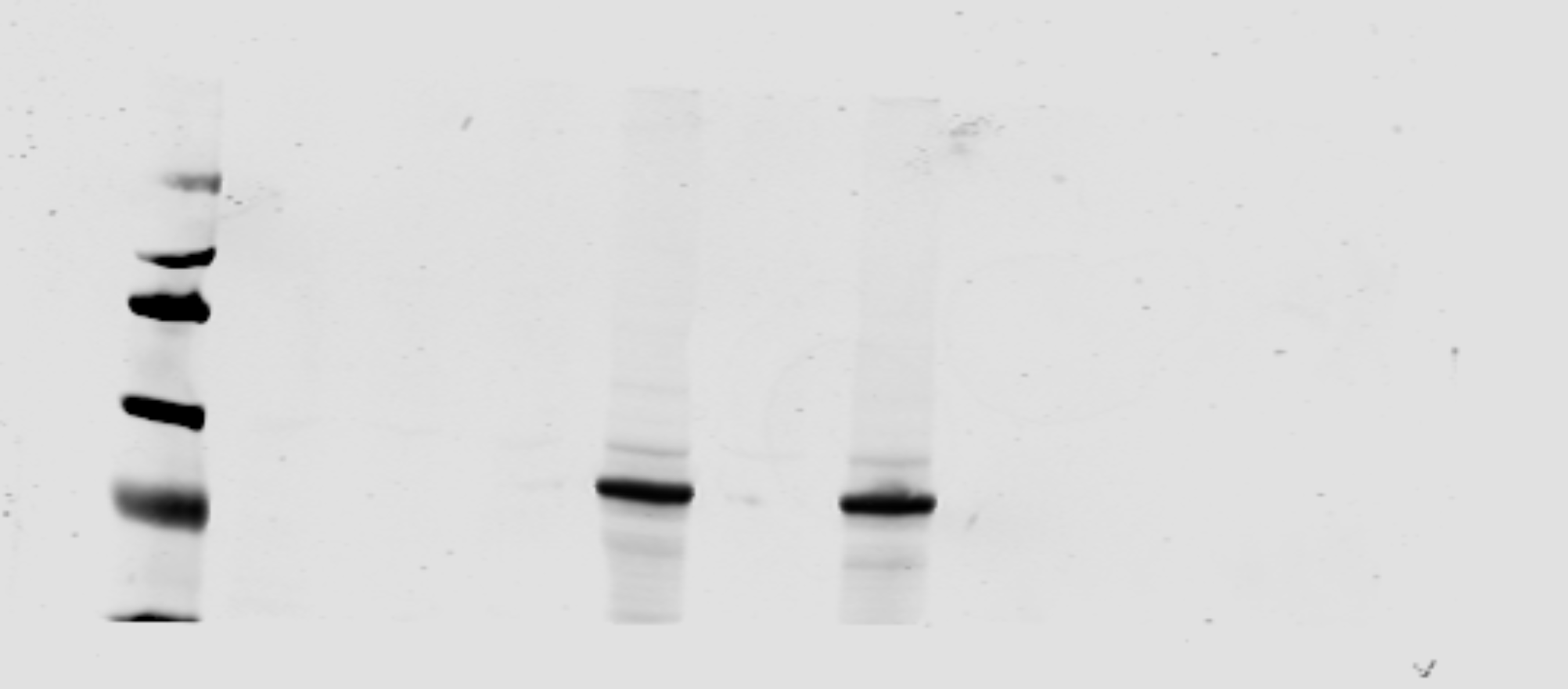

Supplement: Figure 3—source data 1. [file elife-81606-fig3-data1.zip › Figure 3/mx1.tif]

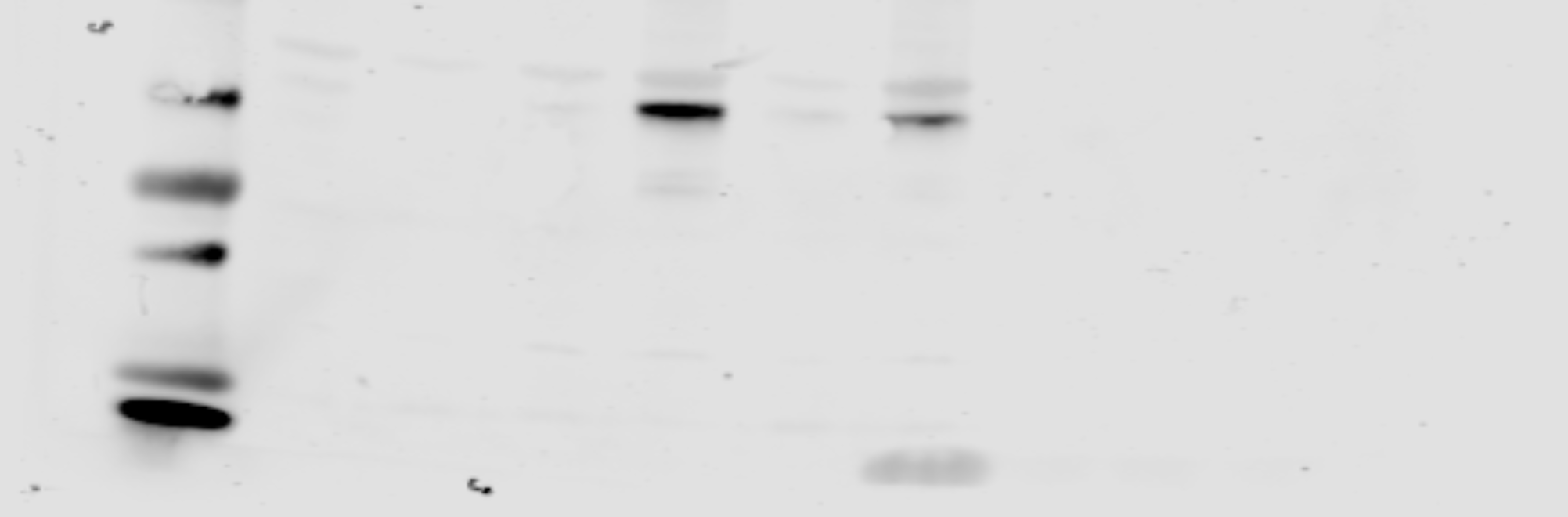

Supplement: Figure 3—source data 1. [file elife-81606-fig3-data1.zip › Figure 3/OAS1.tif]

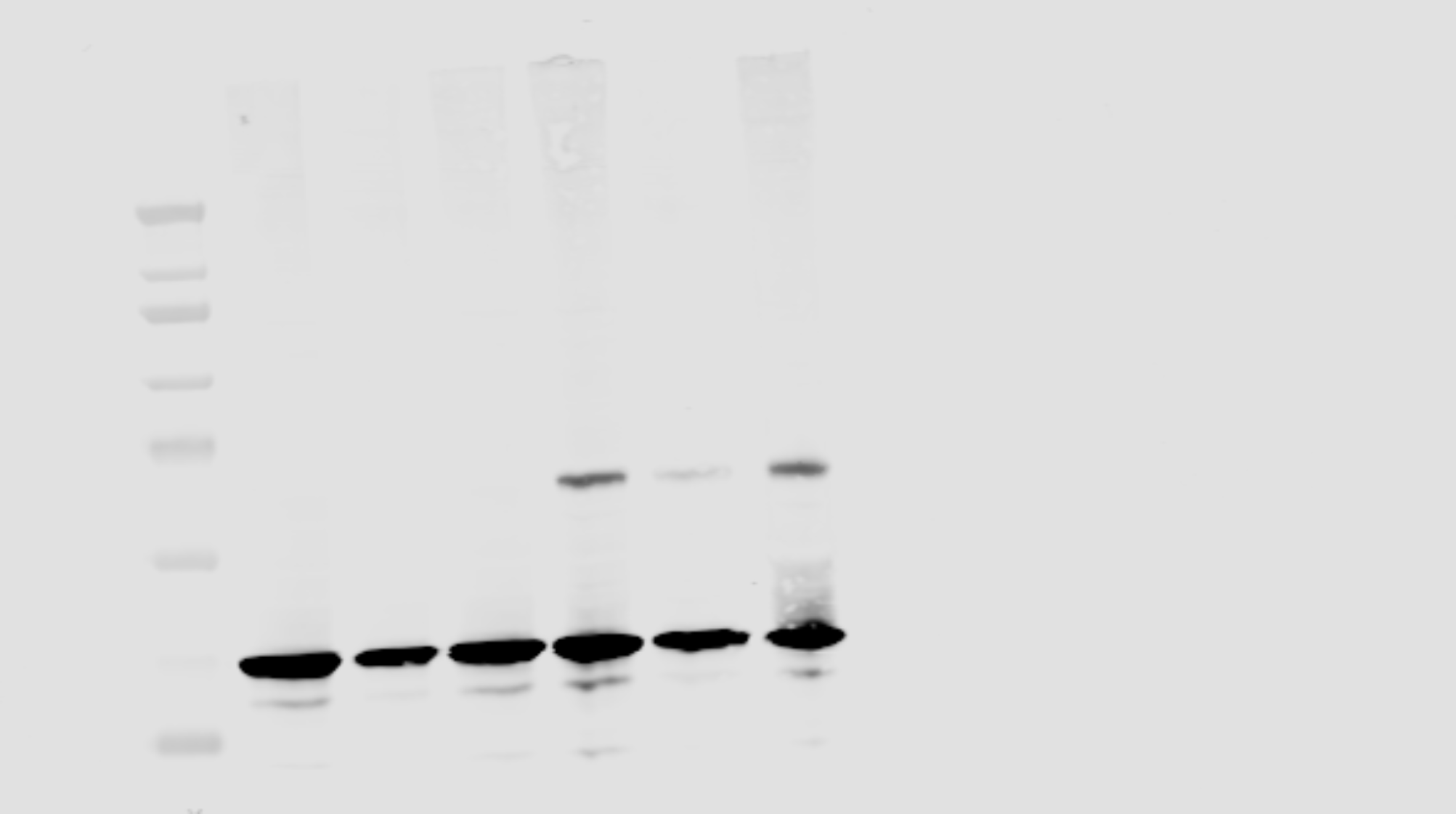

Supplement: Figure 3—source data 1. [file elife-81606-fig3-data1.zip › Figure 3/IFIT3 .tif]

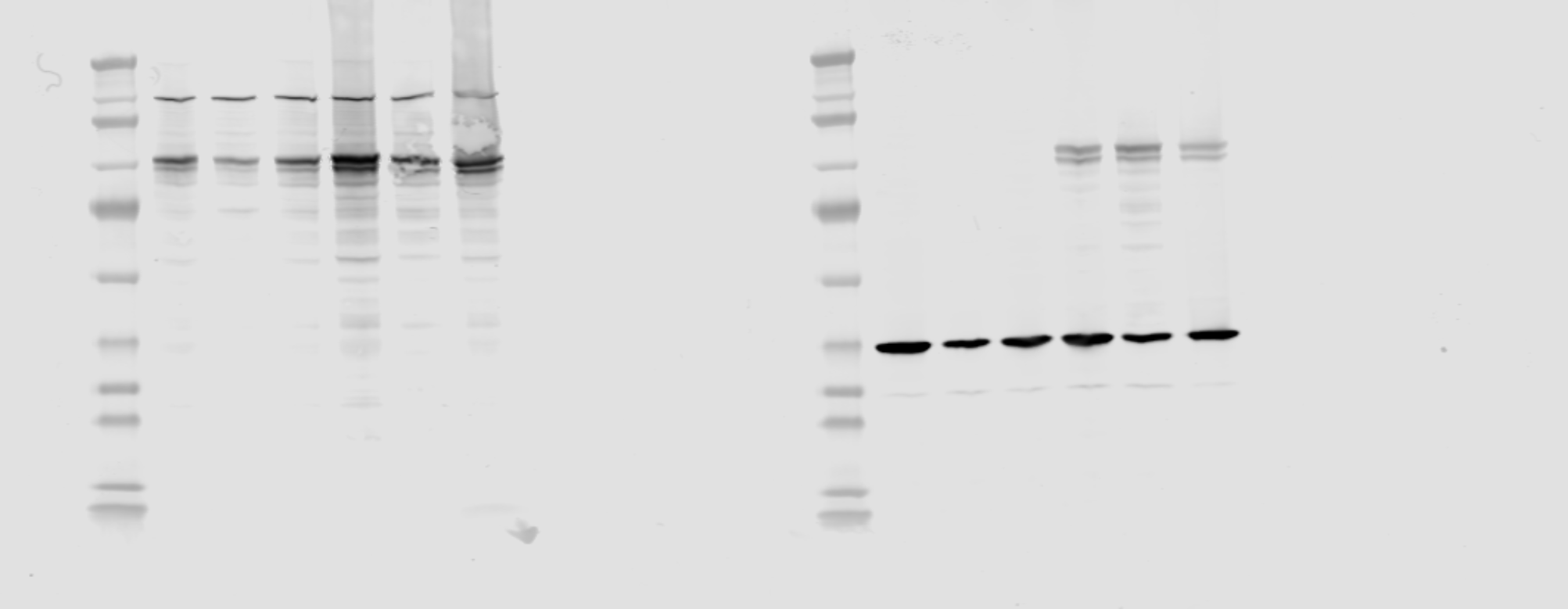

Supplement: Figure 3—source data 1. [file elife-81606-fig3-data1.zip › Figure 3/STAT1 and pSTAT1 and actin .tif]

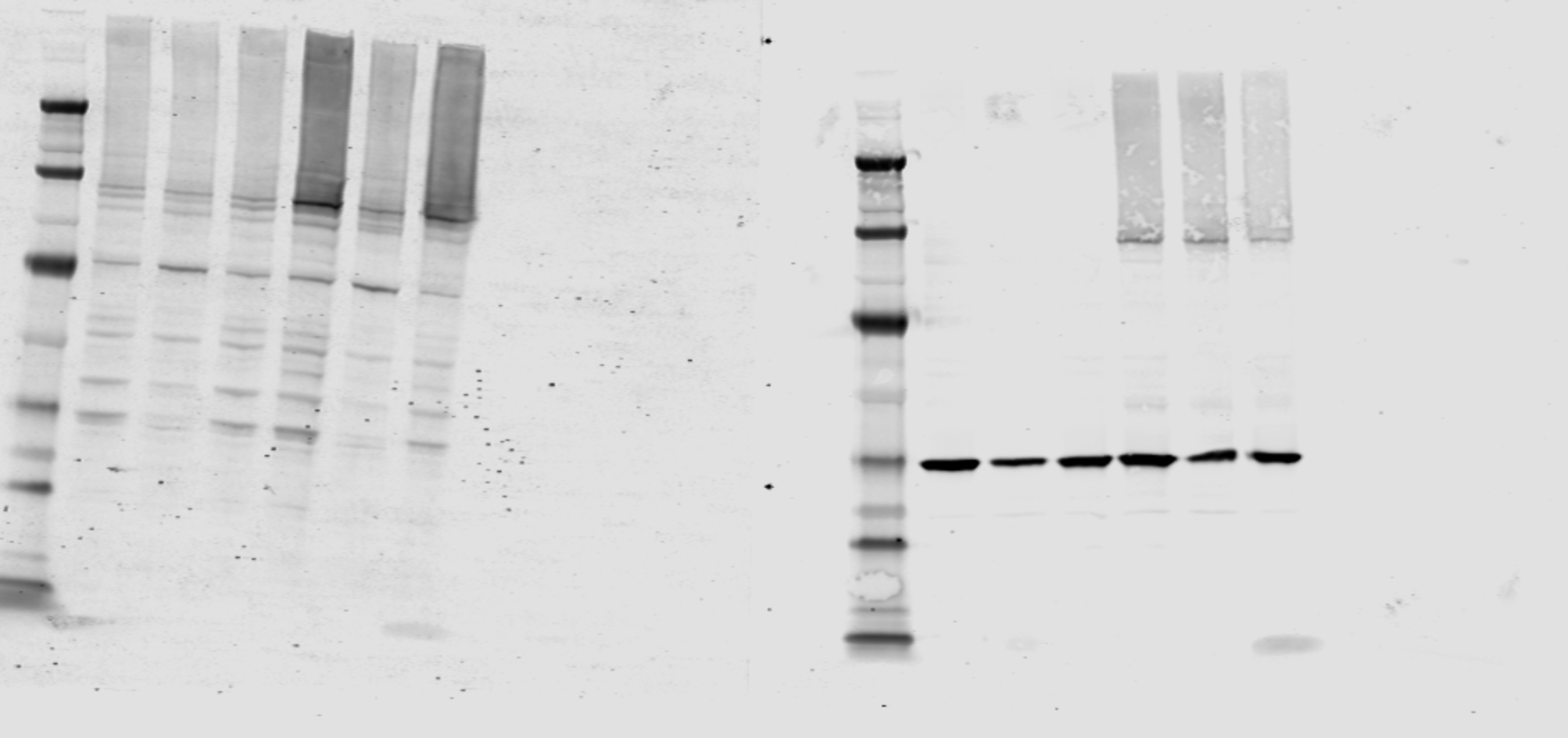

Supplement: Figure 3—source data 1. [file elife-81606-fig3-data1.zip › Figure 3/STAT2 and pSTAT2 and actin.tif]

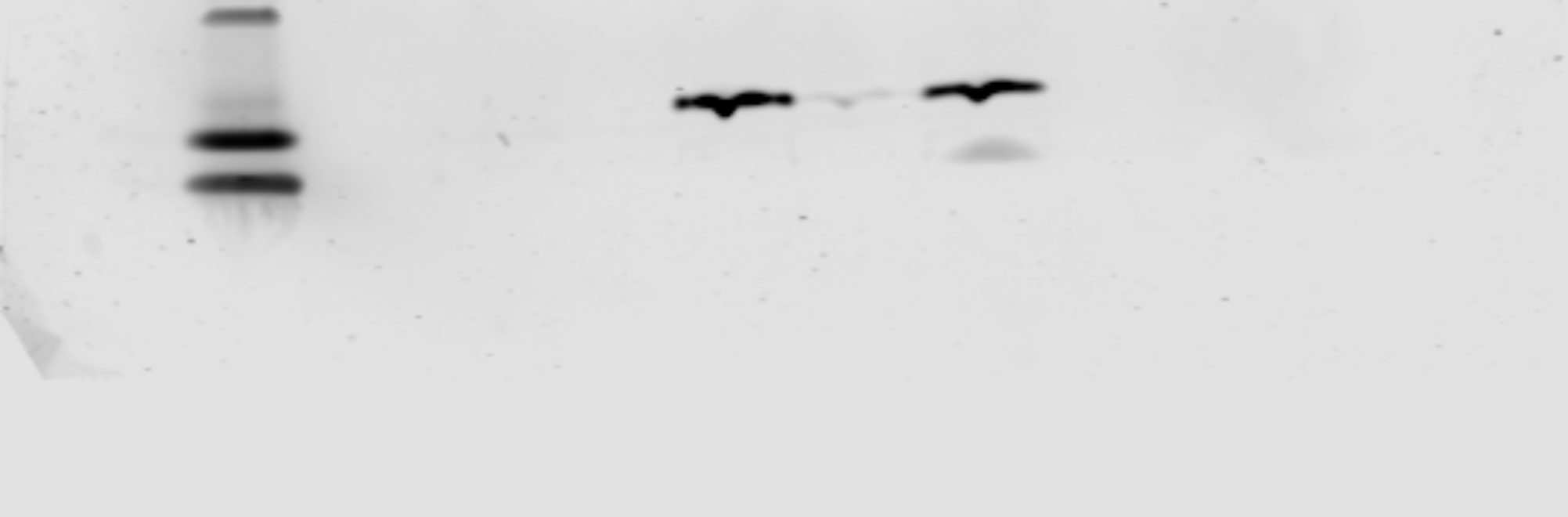

Supplement: Figure 3—source data 1. [file elife-81606-fig3-data1.zip › Figure 3/isg15.tif]

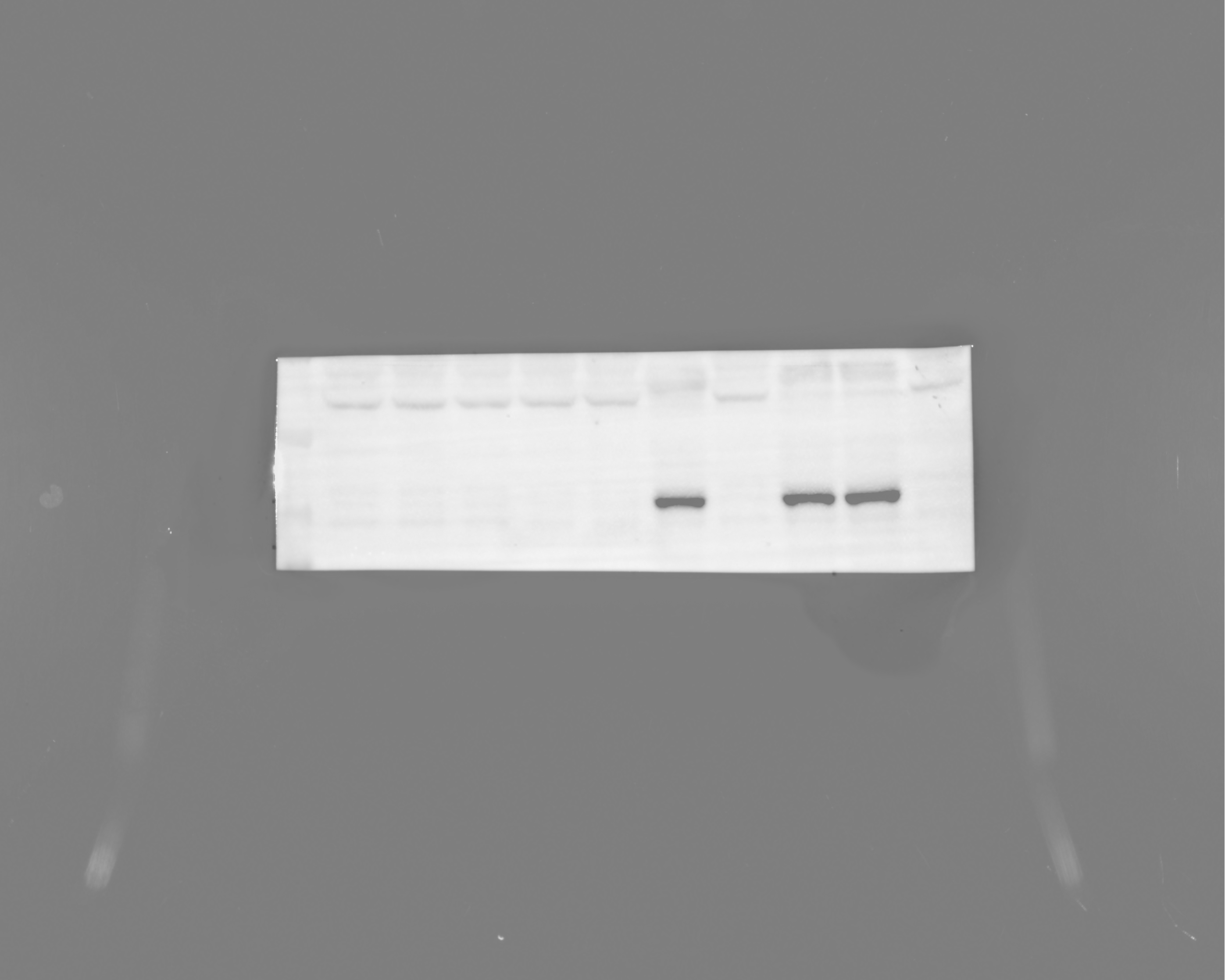

Supplement: Figure 3—figure supplement 1—source data 1. [file elife-81606-fig3-figsupp1-data1.zip › Figure 3 Figure supplement 1/oas1 (Multichannel).tif]

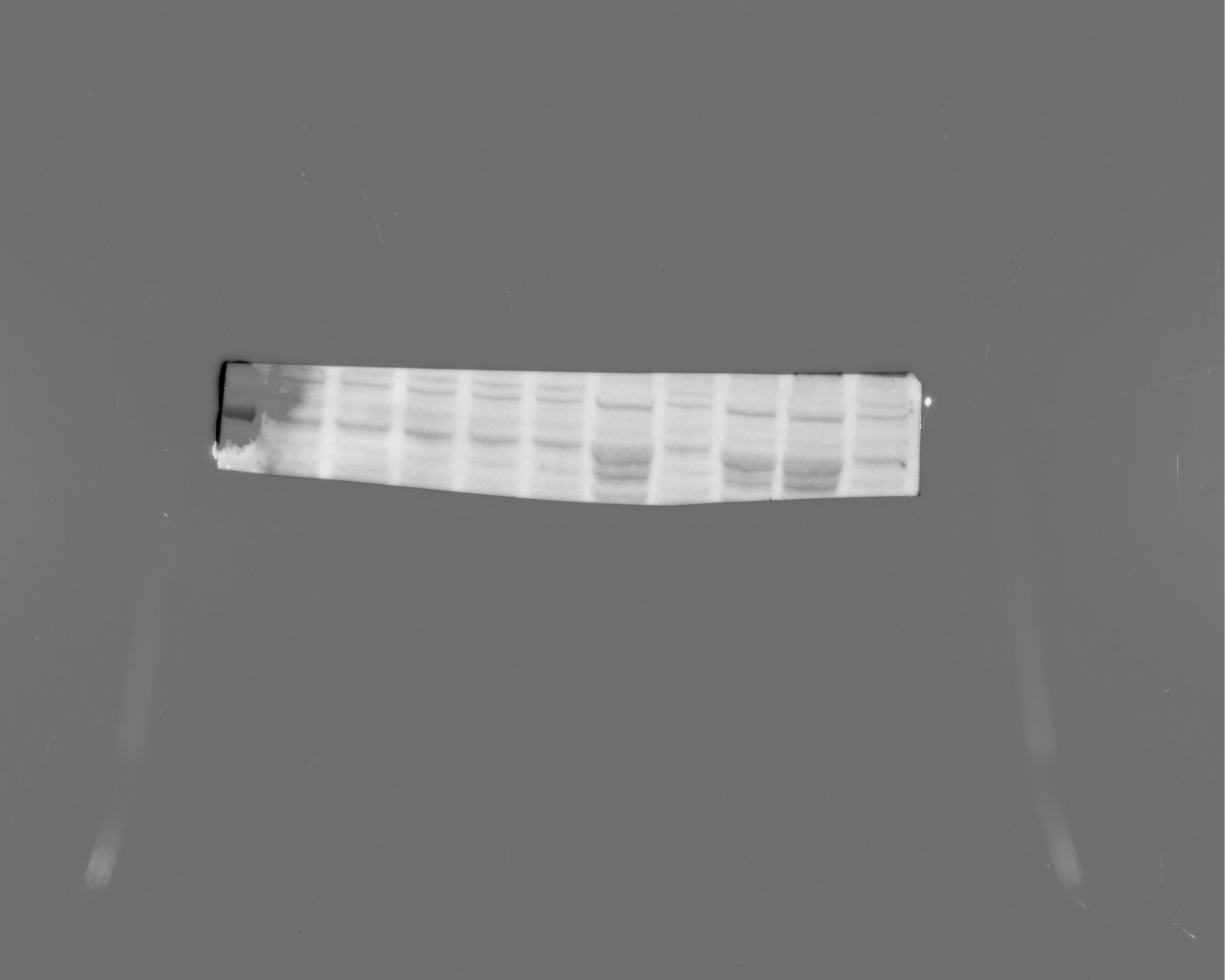

Supplement: Figure 3—figure supplement 1—source data 1. [file elife-81606-fig3-figsupp1-data1.zip › Figure 3 Figure supplement 1/mx1 (Multichannel).tif]

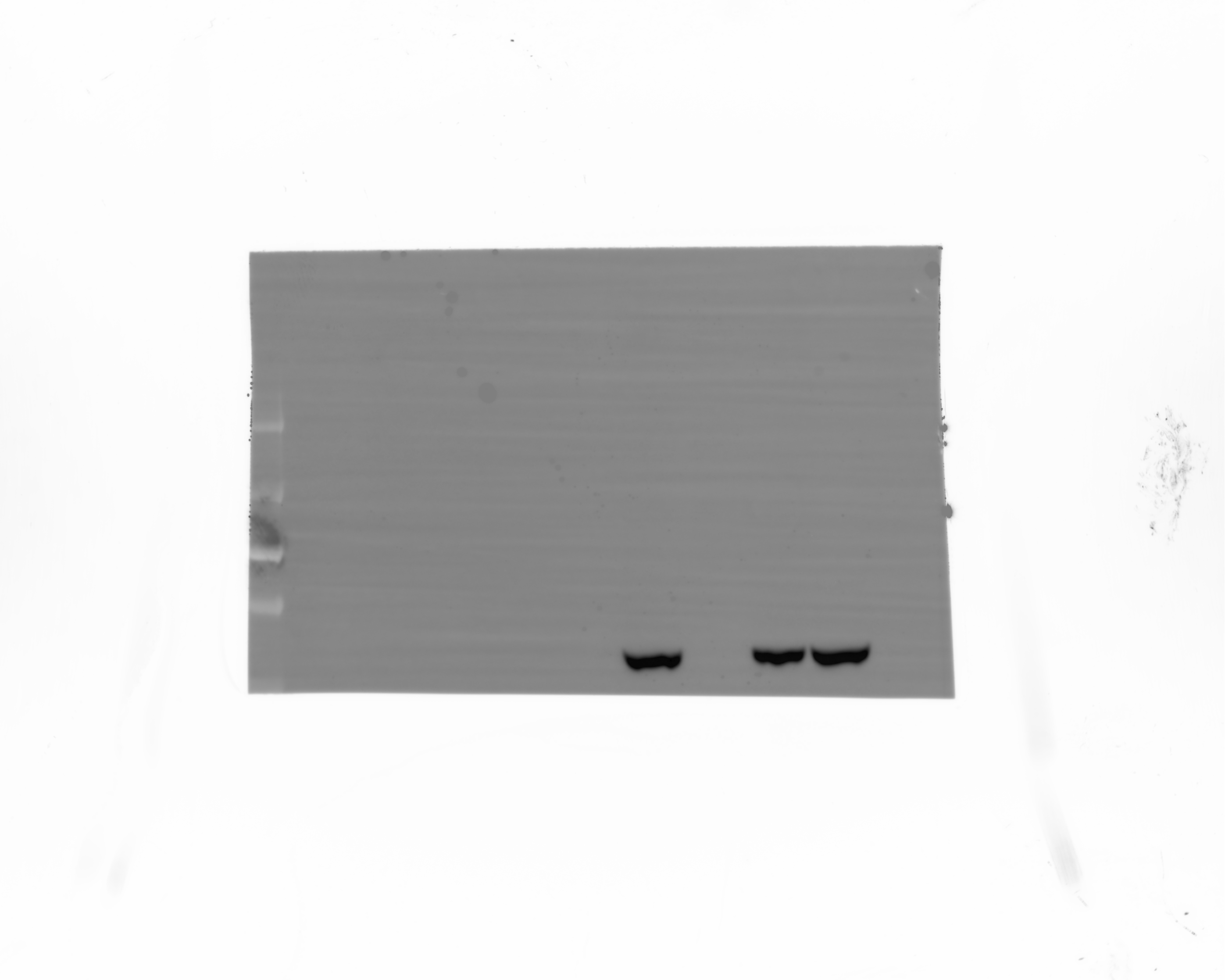

Supplement: Figure 3—figure supplement 1—source data 1. [file elife-81606-fig3-figsupp1-data1.zip › Figure 3 Figure supplement 1/ifit3 (Multichannel).tif]

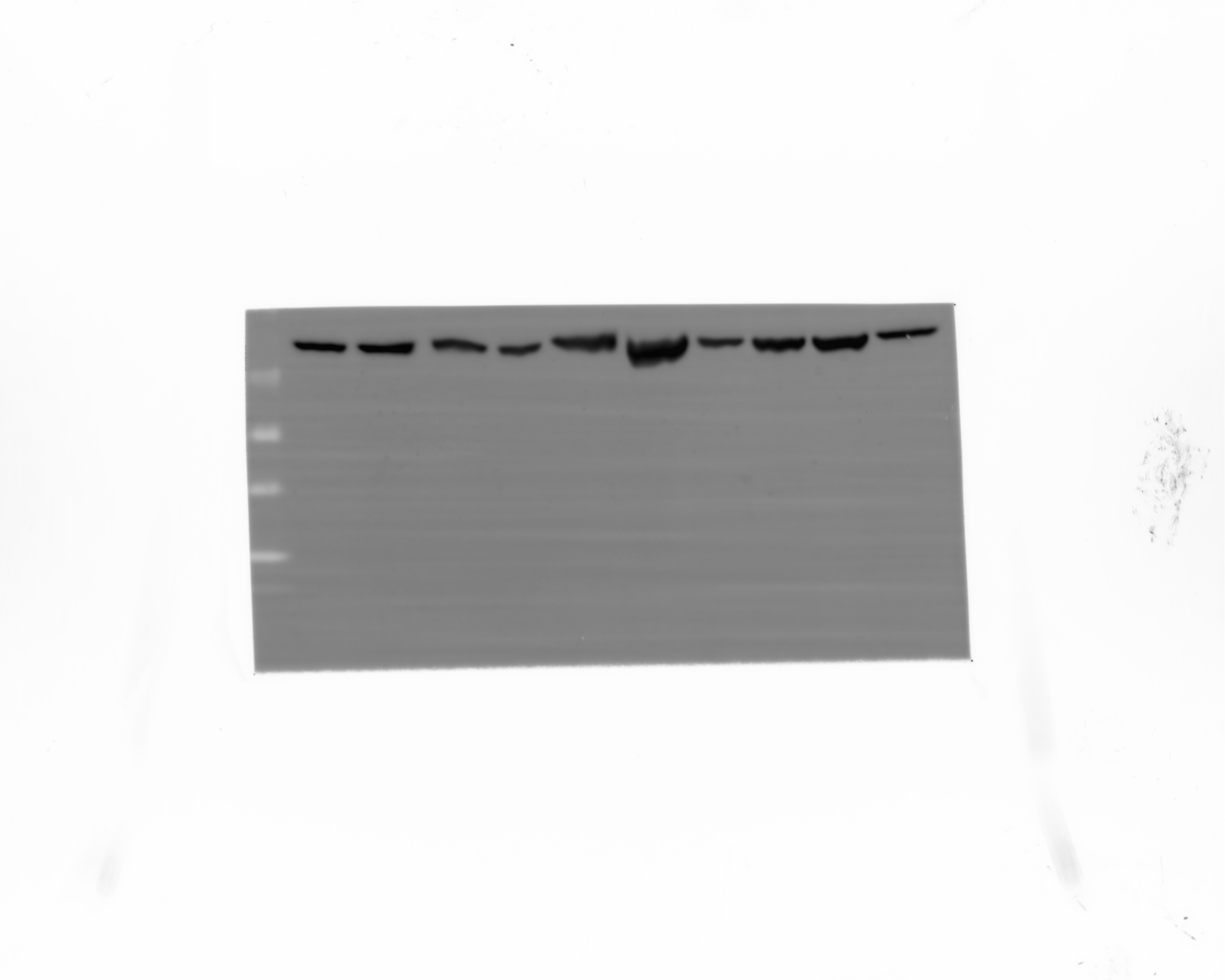

Supplement: Figure 3—figure supplement 1—source data 1. [file elife-81606-fig3-figsupp1-data1.zip › Figure 3 Figure supplement 1/b actin (Multichannel).tif]

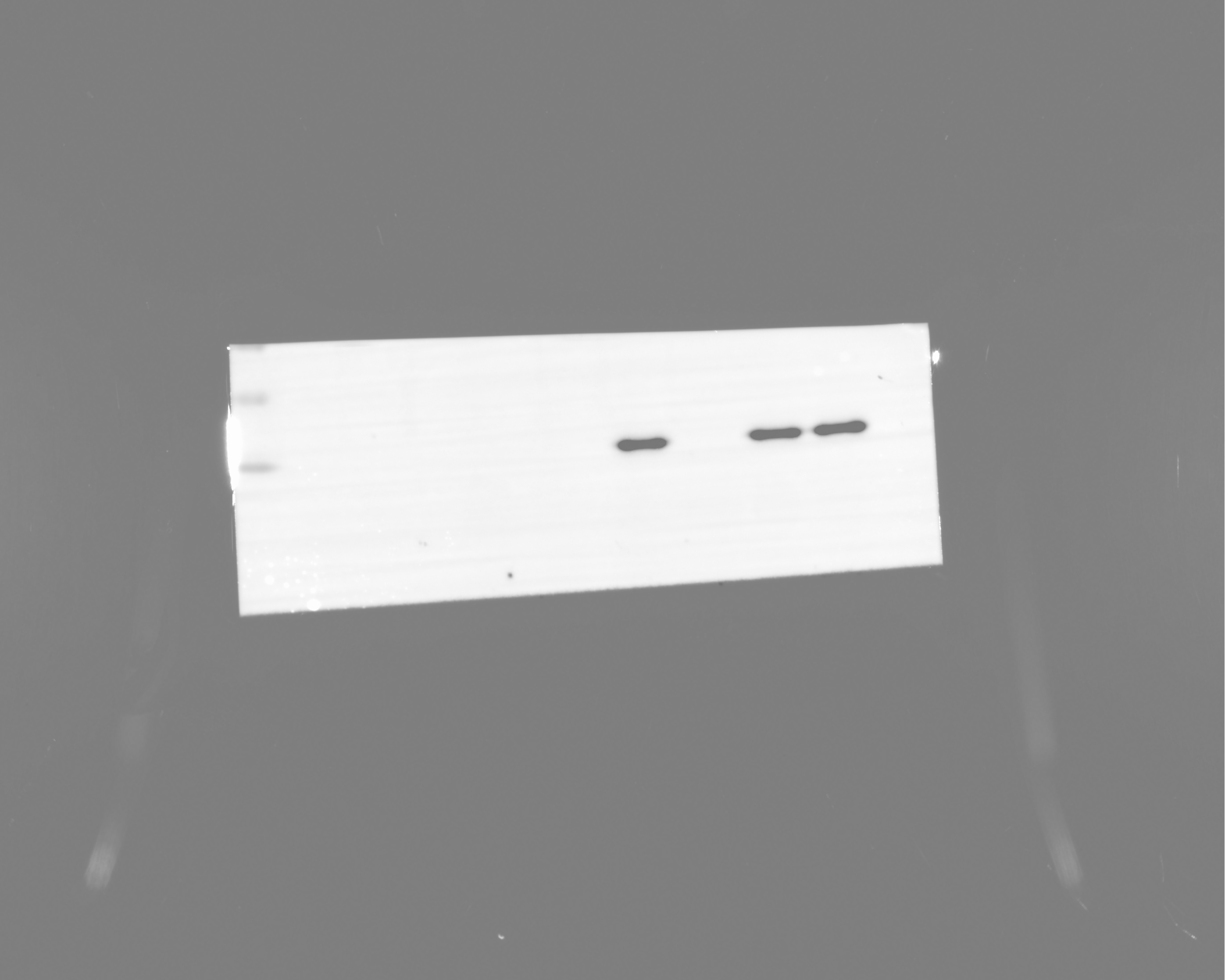

Supplement: Figure 3—figure supplement 1—source data 1. [file elife-81606-fig3-figsupp1-data1.zip › Figure 3 Figure supplement 1/isg15 (Multichannel).tif]

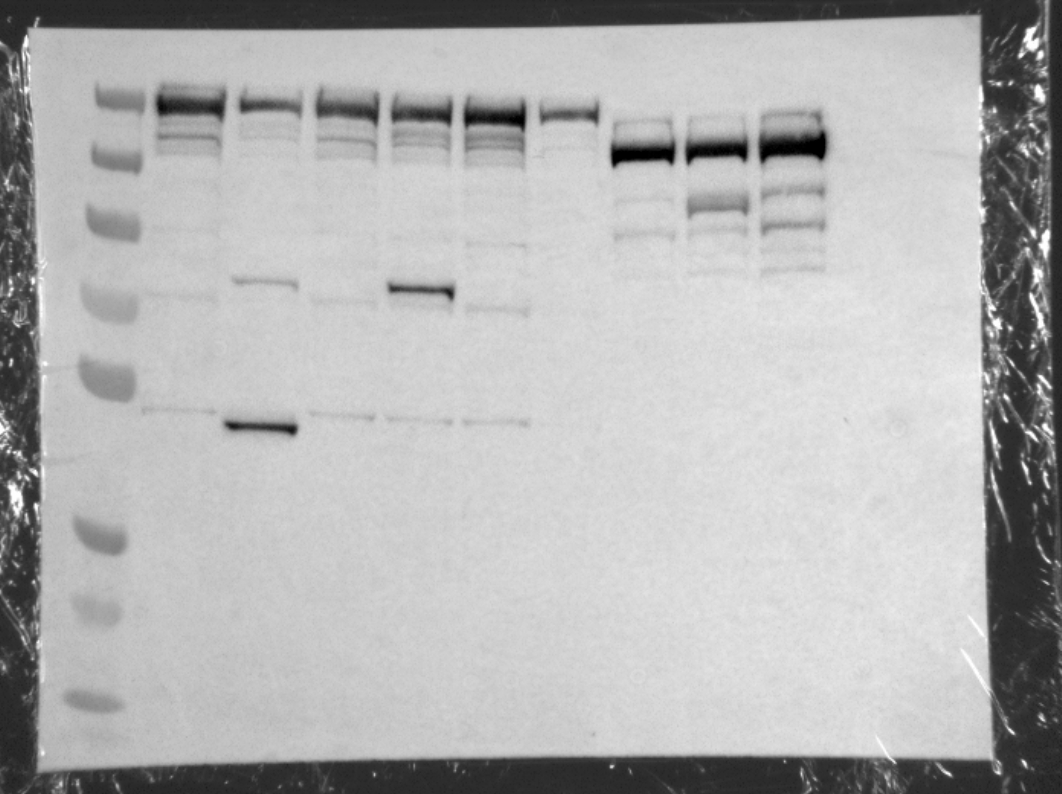

Supplement: Figure 5—source data 1. [file elife-81606-fig5-data1.zip › Figure 5-source data 1/figure5d_Myc_merged.jpg]

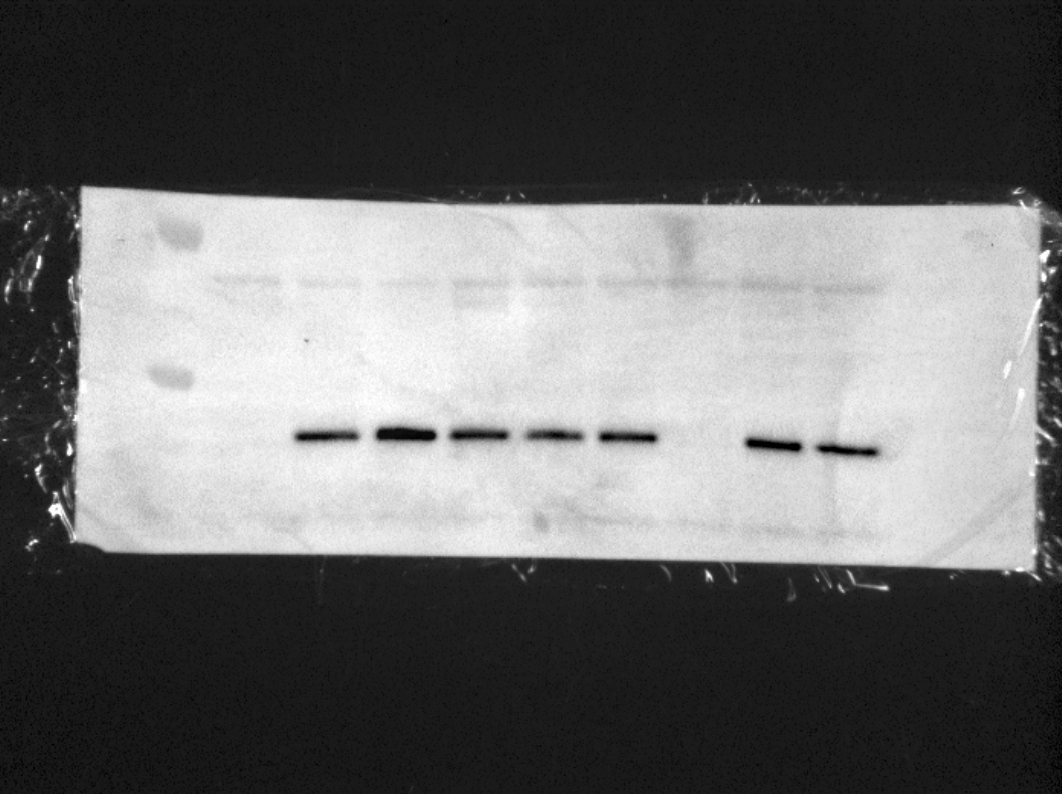

Supplement: Figure 5—source data 1. [file elife-81606-fig5-data1.zip › Figure 5-source data 1/figure5c_HA_merged.jpg]

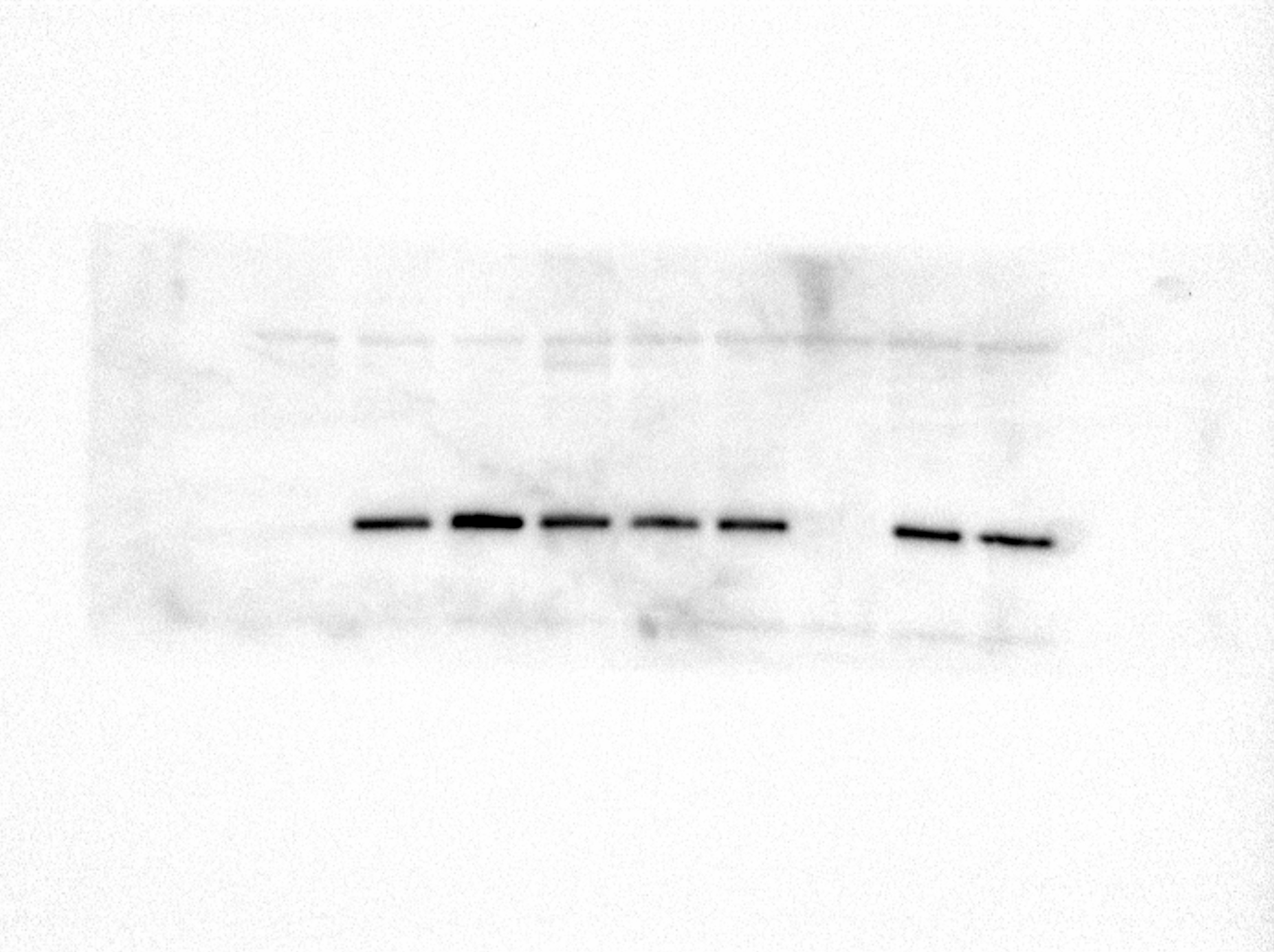

Supplement: Figure 5—source data 1. [file elife-81606-fig5-data1.zip › Figure 5-source data 1/figure5c_HA.jpg]

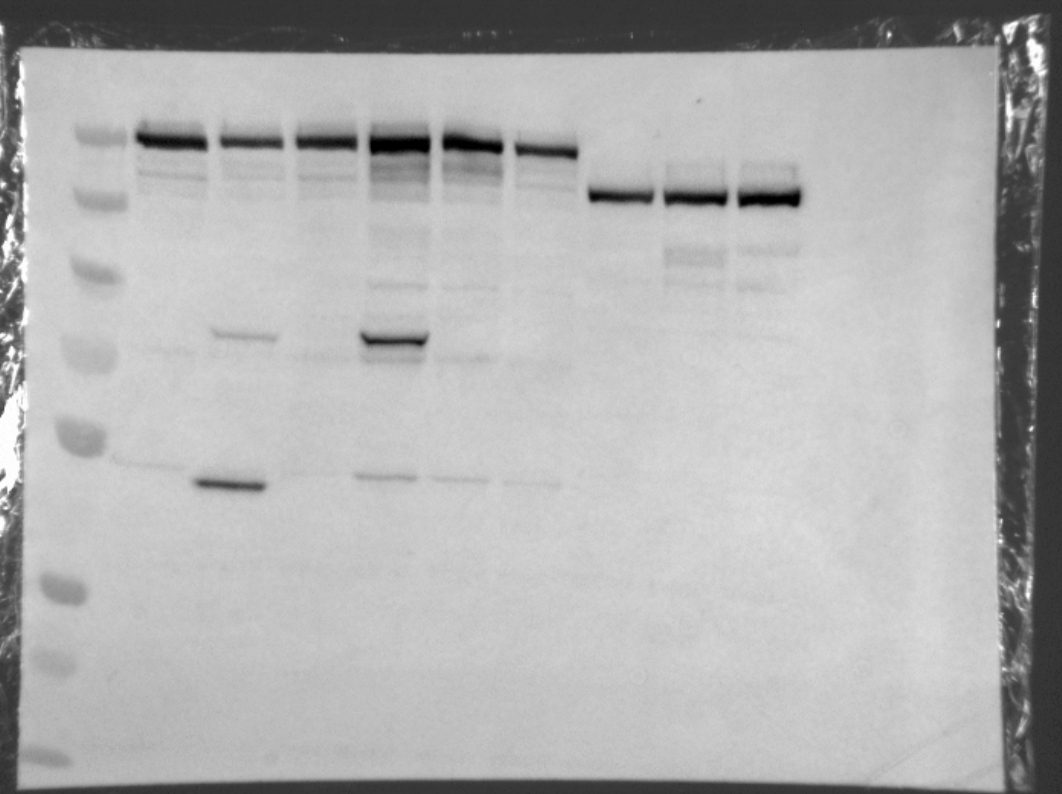

Supplement: Figure 5—source data 1. [file elife-81606-fig5-data1.zip › Figure 5-source data 1/figure5c_Myc_merged.jpg]

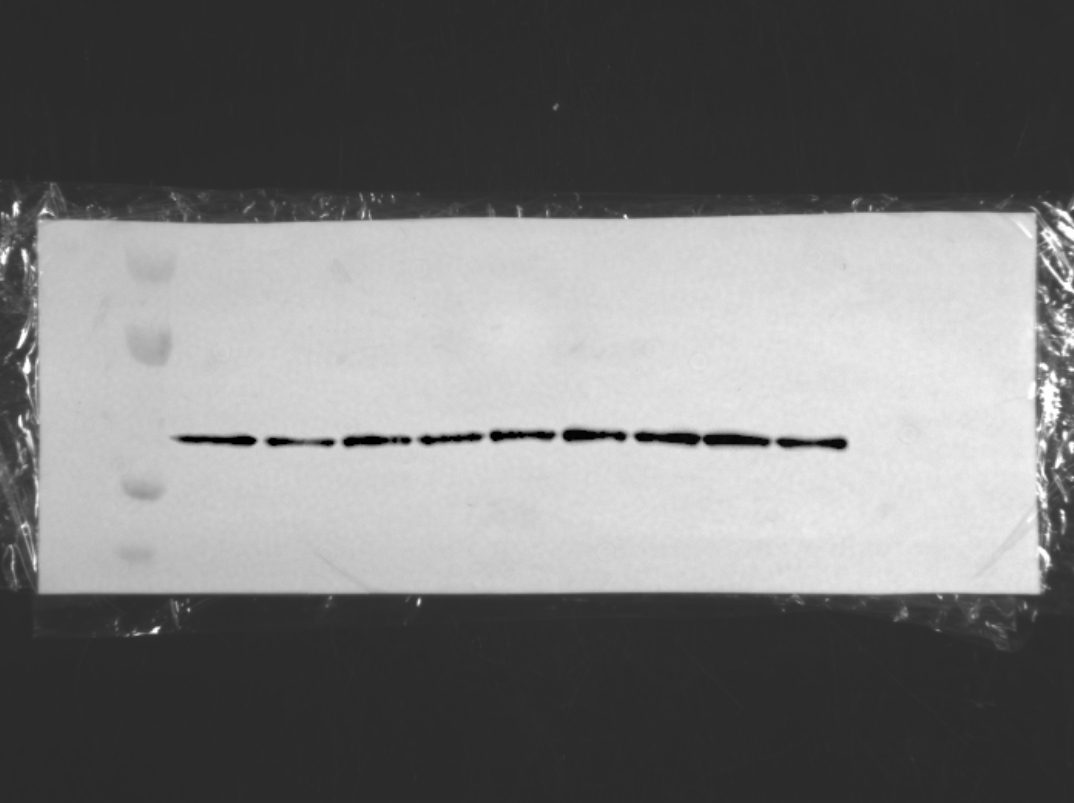

Supplement: Figure 5—source data 1. [file elife-81606-fig5-data1.zip › Figure 5-source data 1/figure5d_GAPDH_merged.jpg]

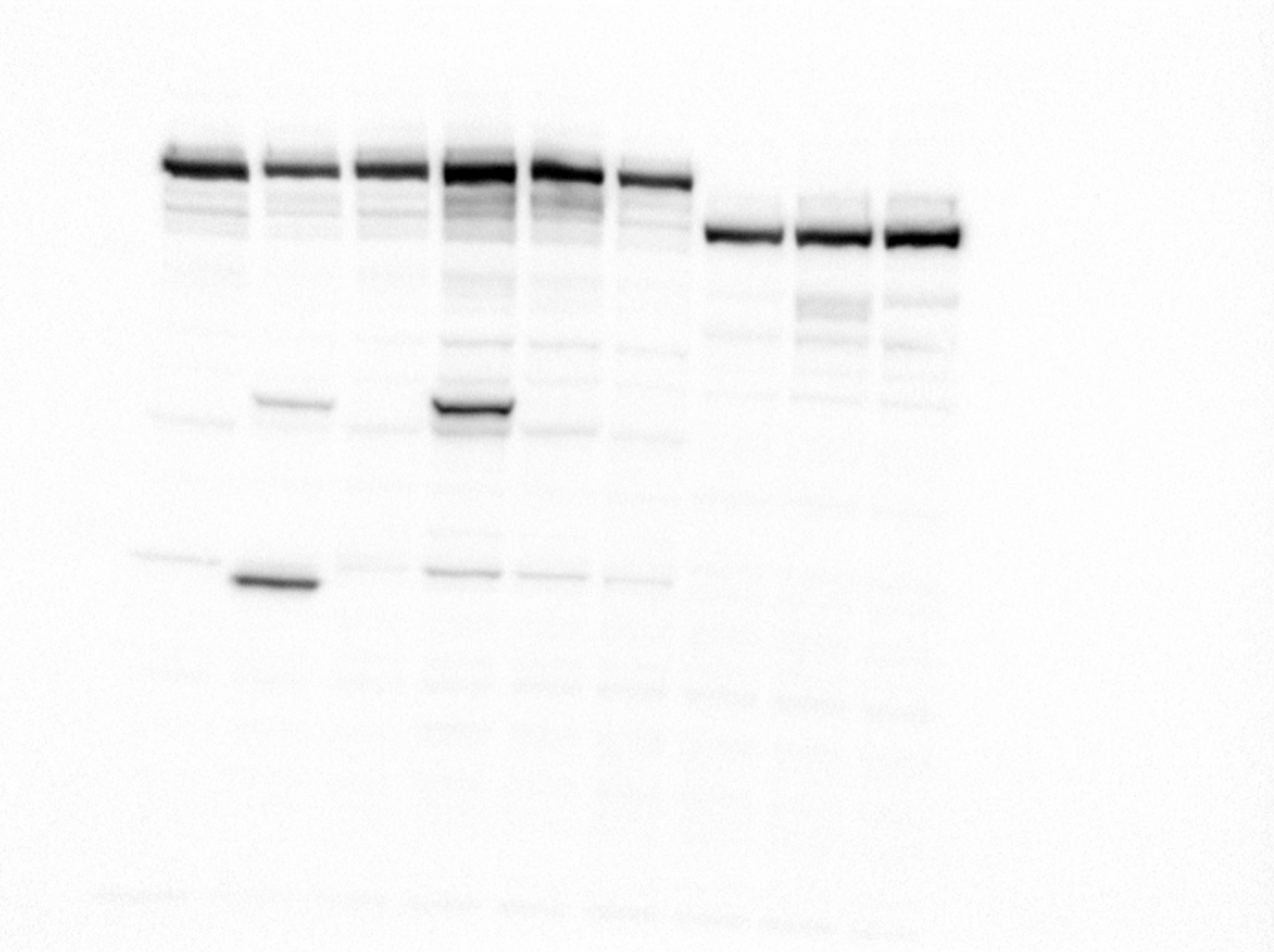

Supplement: Figure 5—source data 1. [file elife-81606-fig5-data1.zip › Figure 5-source data 1/figure5c_Myc.jpg]

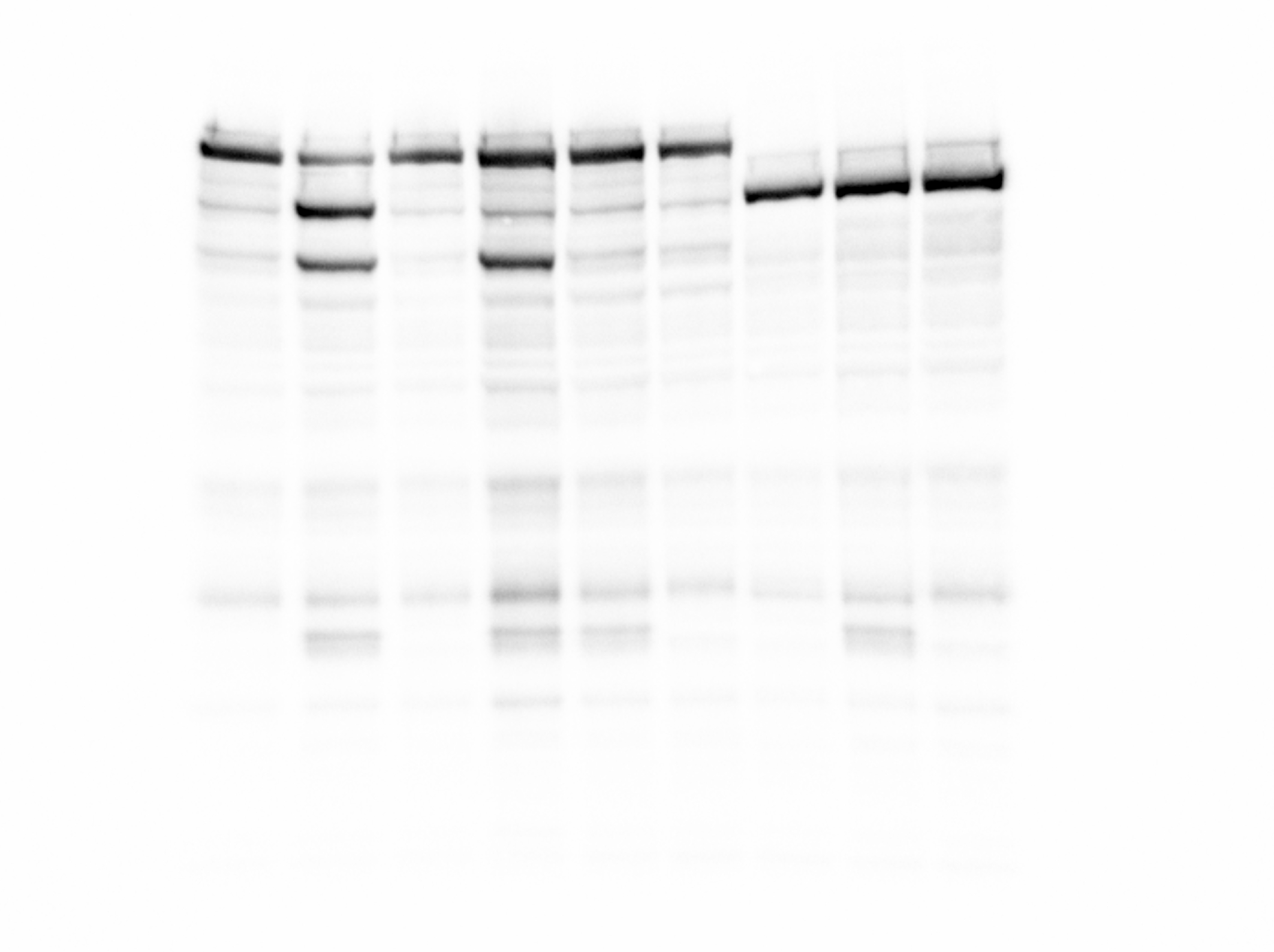

Supplement: Figure 5—source data 1. [file elife-81606-fig5-data1.zip › Figure 5-source data 1/figure5c_FLAG.jpg]

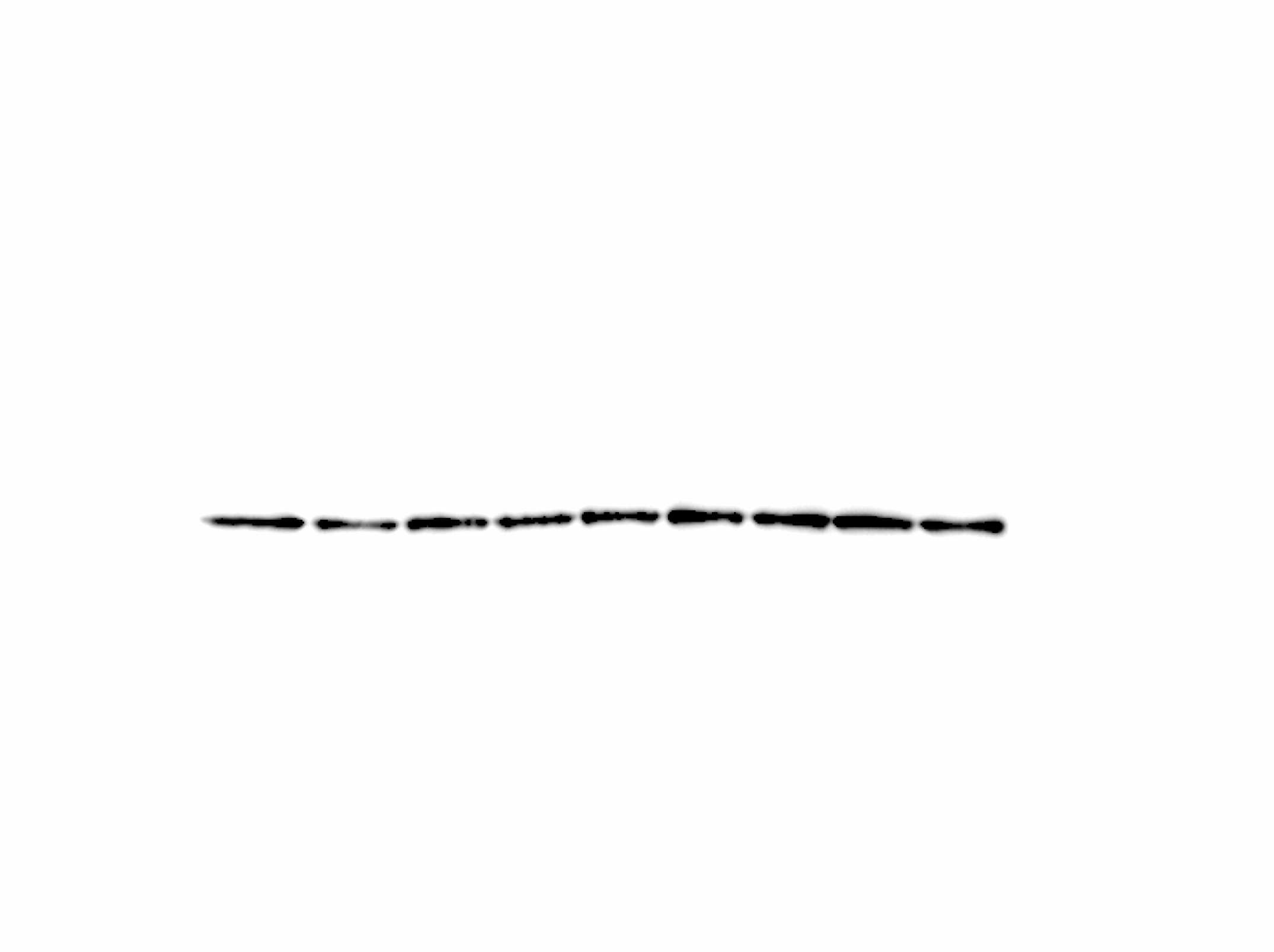

Supplement: Figure 5—source data 1. [file elife-81606-fig5-data1.zip › Figure 5-source data 1/figure5d_GAPDH.jpg]

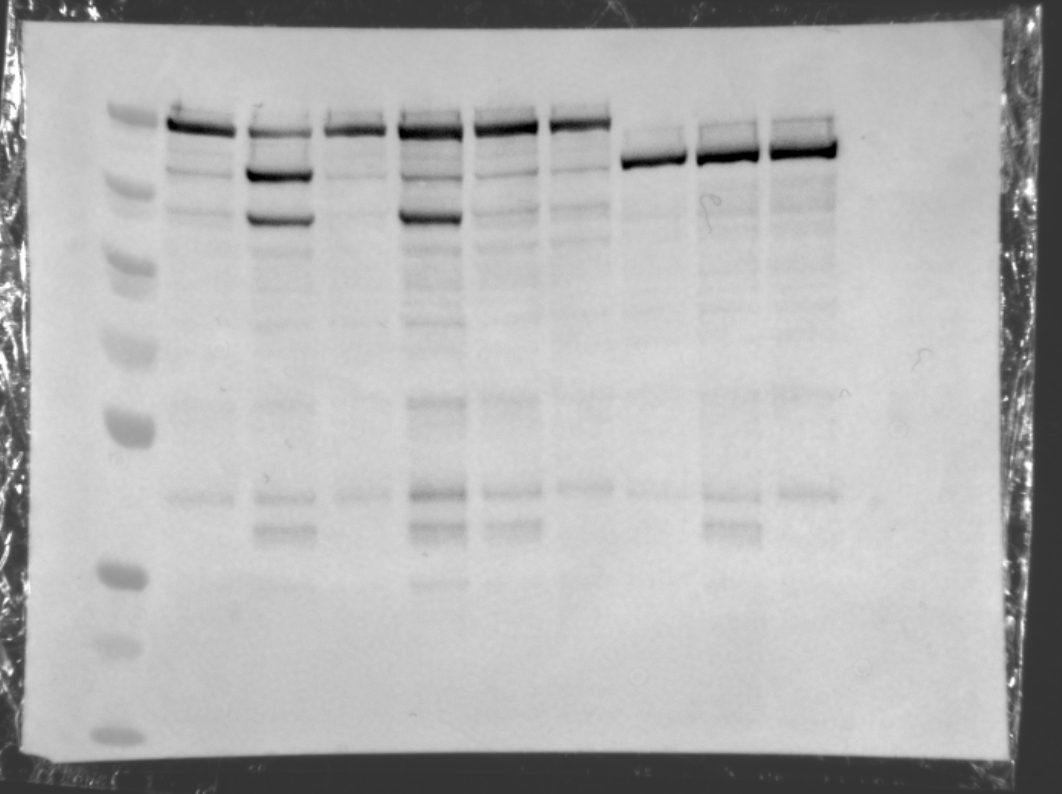

Supplement: Figure 5—source data 1. [file elife-81606-fig5-data1.zip › Figure 5-source data 1/figure5c_FLAG_merged.jpg]

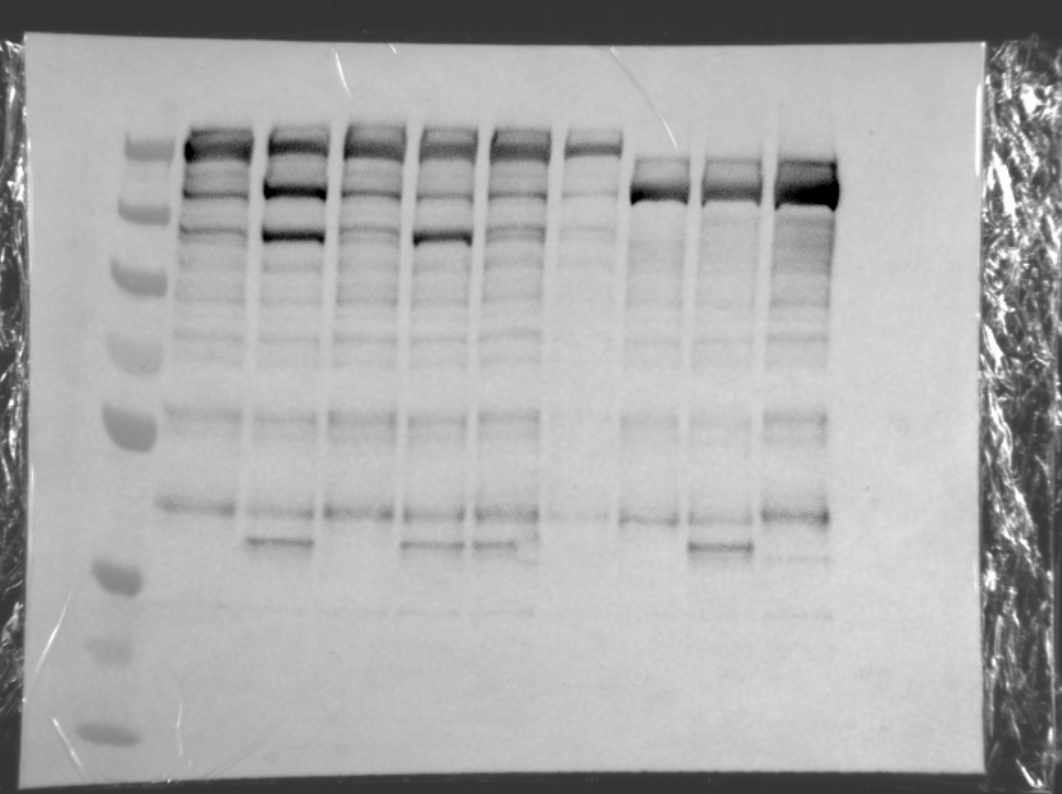

Supplement: Figure 5—source data 1. [file elife-81606-fig5-data1.zip › Figure 5-source data 1/figure5d_FLAG_merged.jpg]

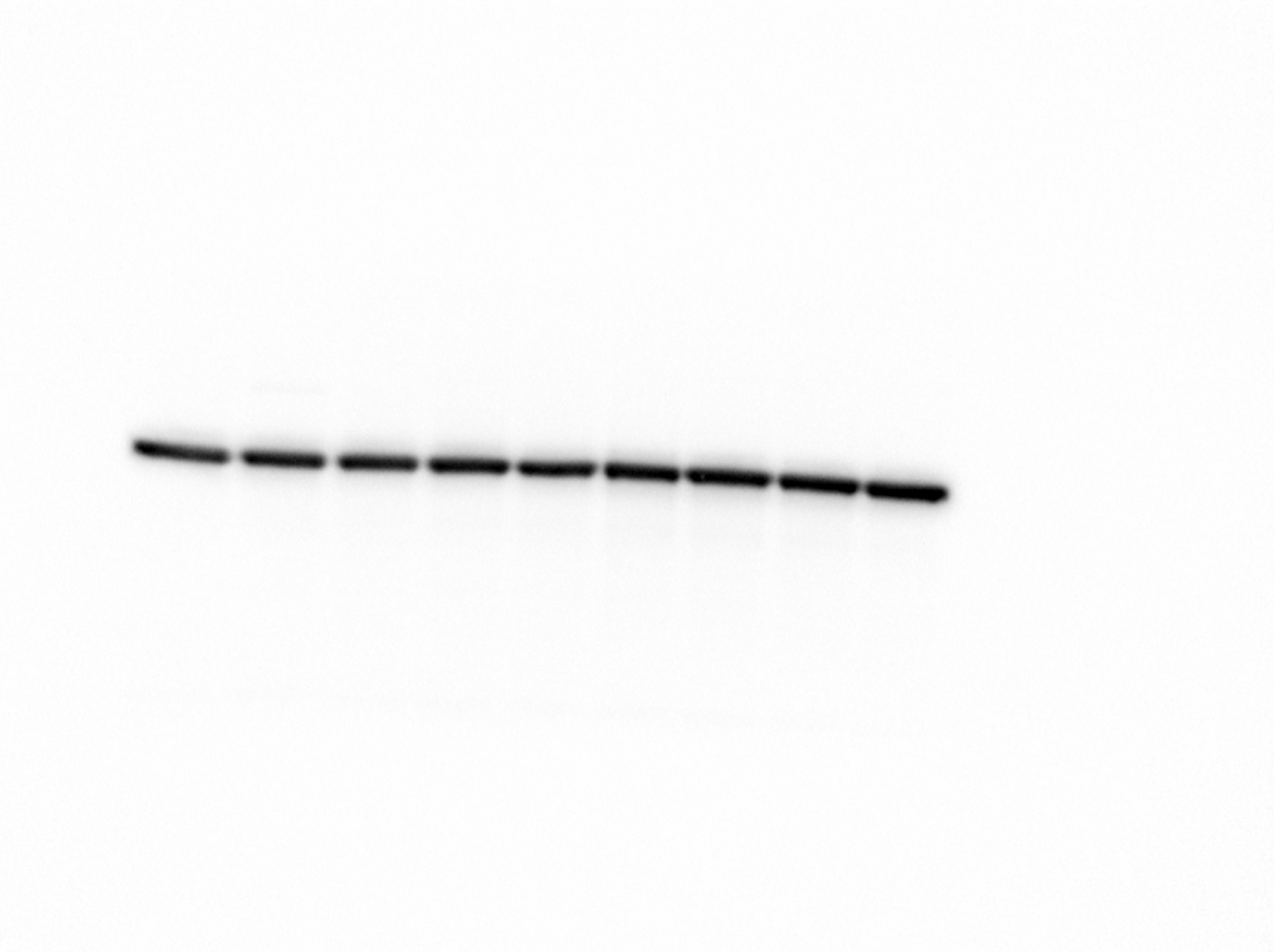

Supplement: Figure 5—source data 1. [file elife-81606-fig5-data1.zip › Figure 5-source data 1/figure5c_GAPDH.jpg]

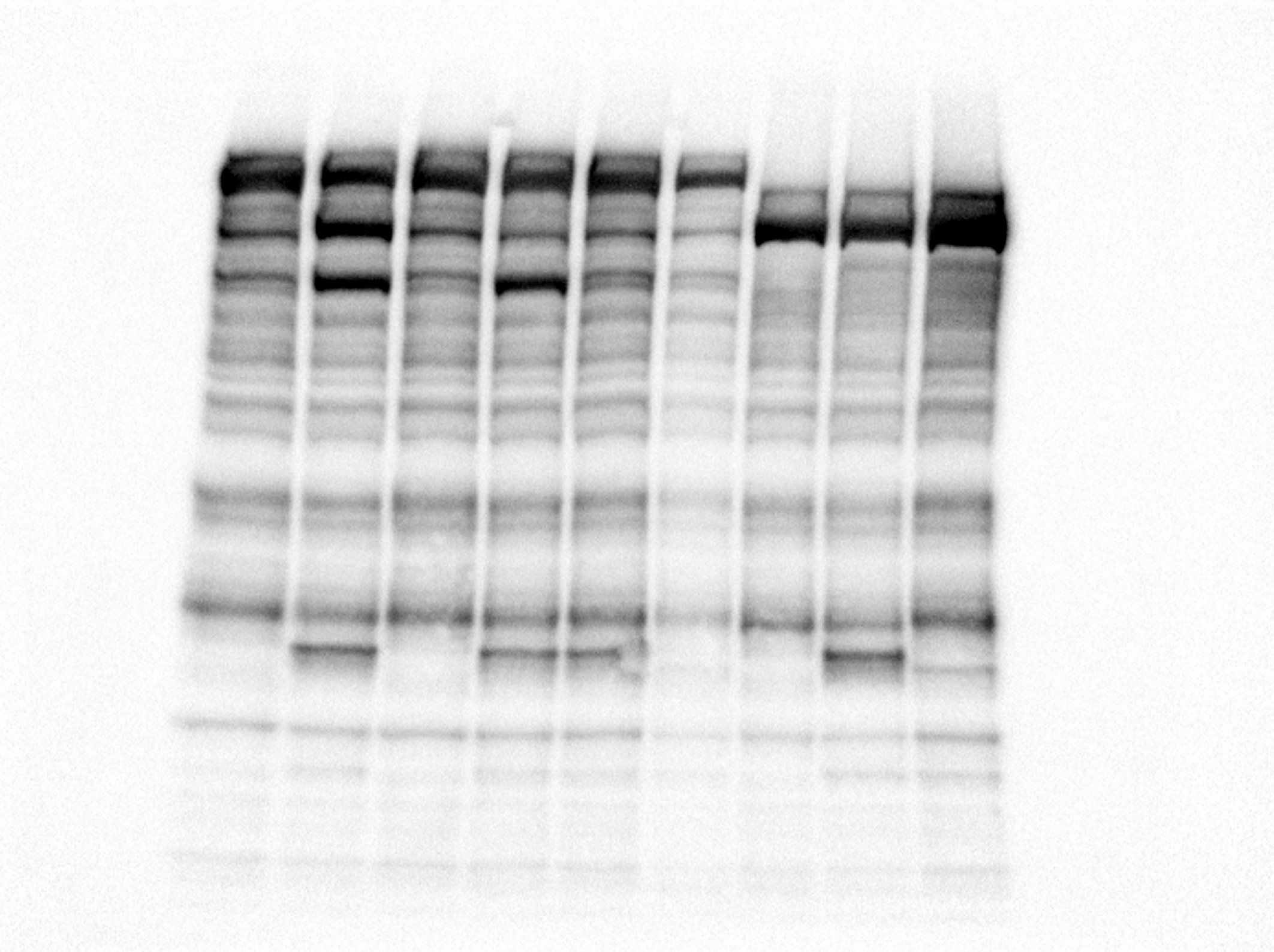

Supplement: Figure 5—source data 1. [file elife-81606-fig5-data1.zip › Figure 5-source data 1/figure5d_FLAG.jpg]

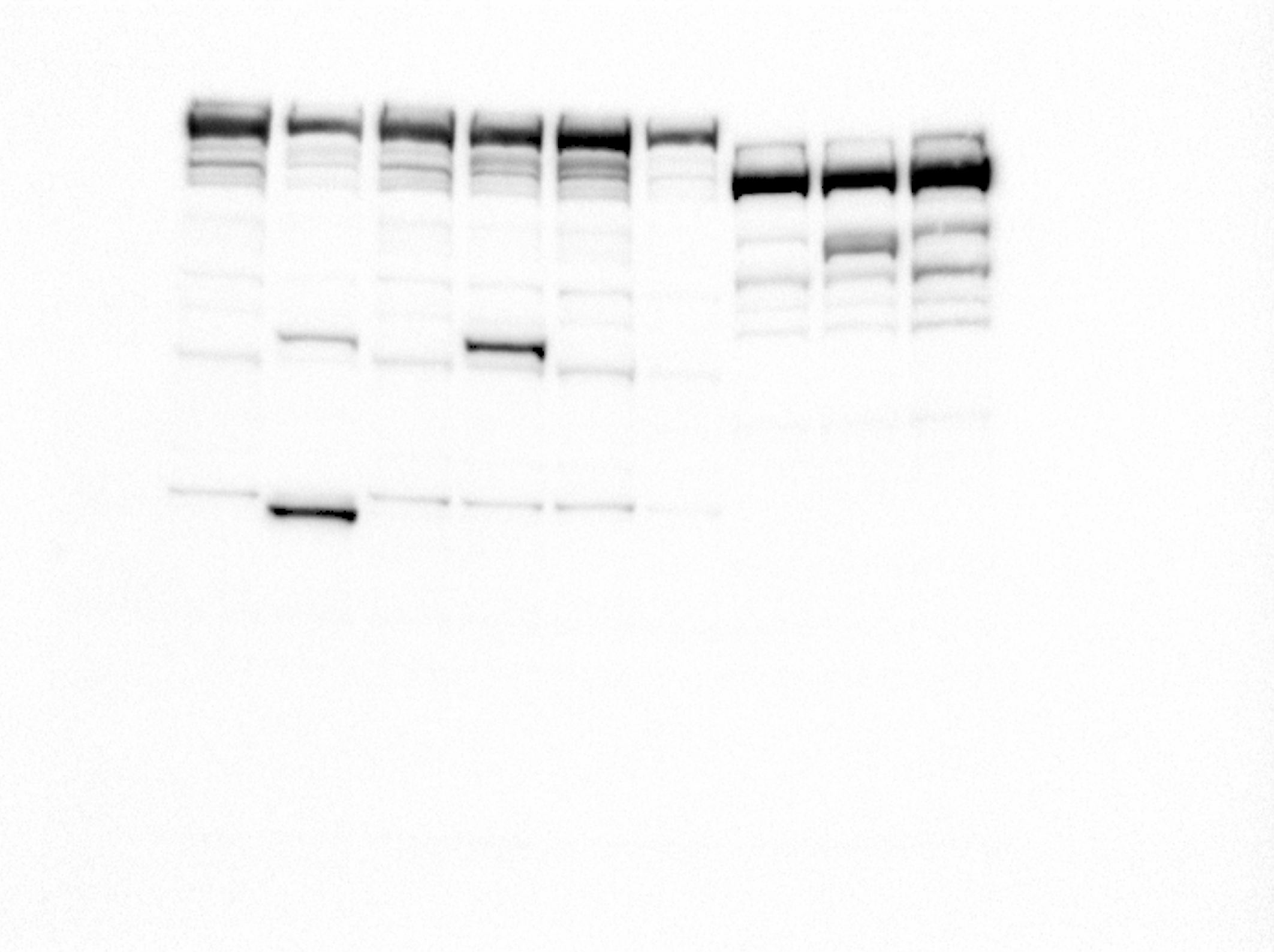

Supplement: Figure 5—source data 1. [file elife-81606-fig5-data1.zip › Figure 5-source data 1/figure5d_Myc.jpg]

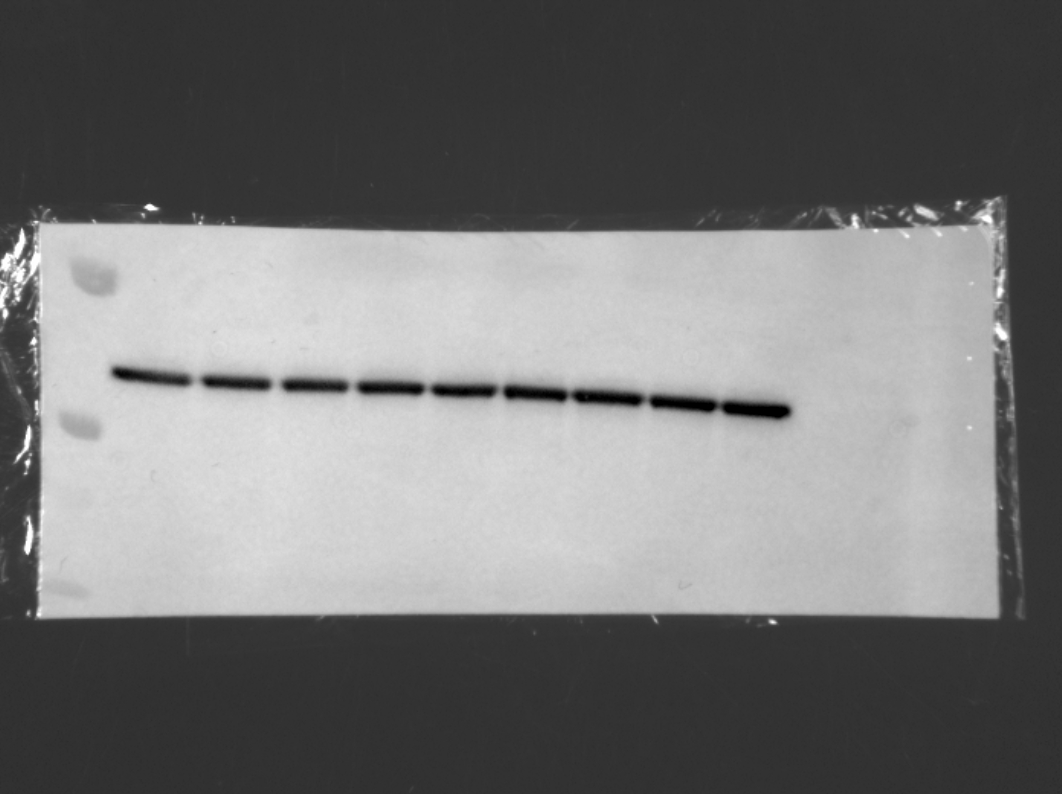

Supplement: Figure 5—source data 1. [file elife-81606-fig5-data1.zip › Figure 5-source data 1/figure5c_GAPDH_merge.jpg]

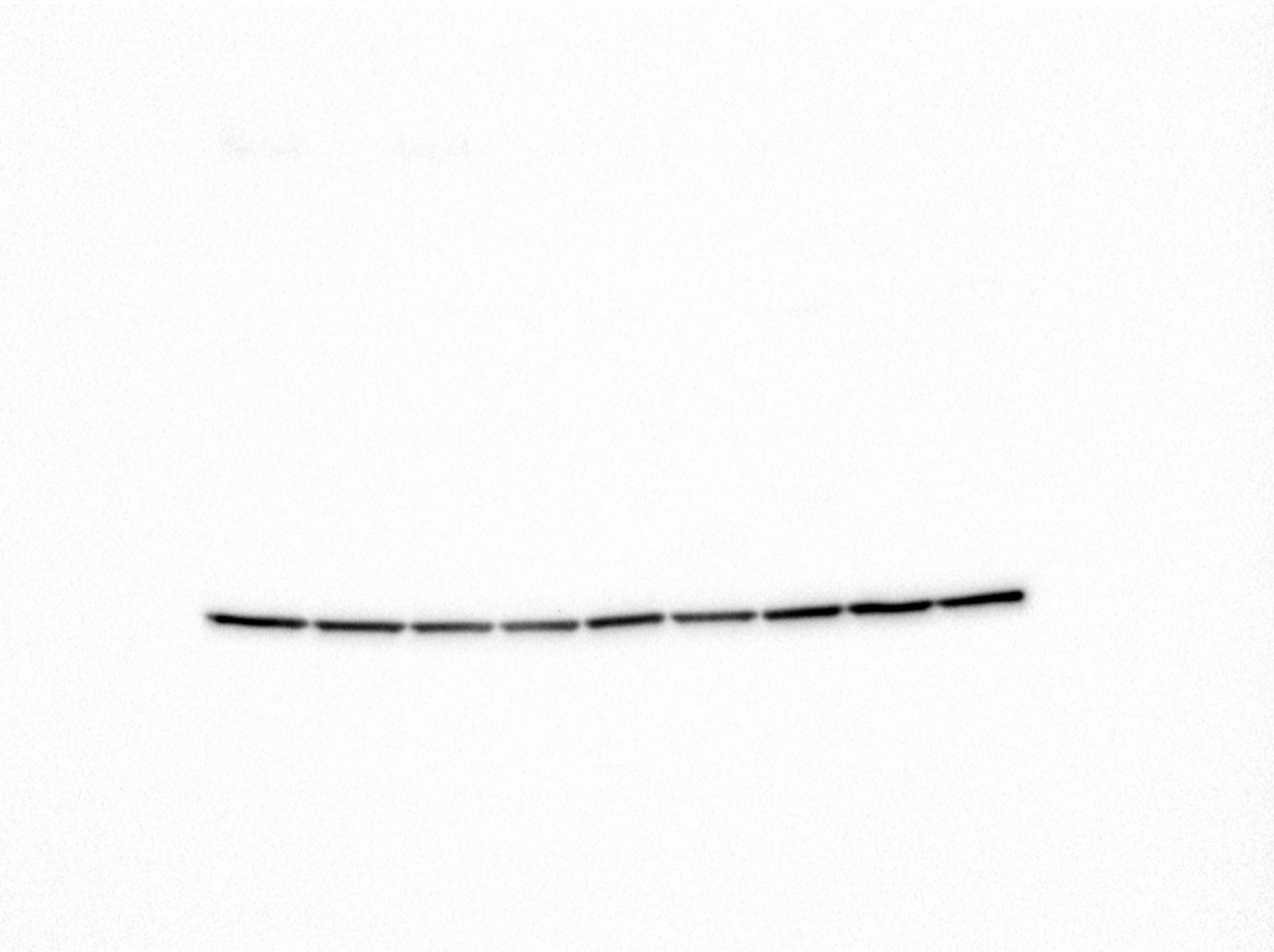

Supplement: Figure 5—figure supplement 1—source data 1. [file elife-81606-fig5-figsupp1-data1.zip › Figure 5-Figure supplement 1-source data /figure5-supplement1B_GAPDH.jpg]

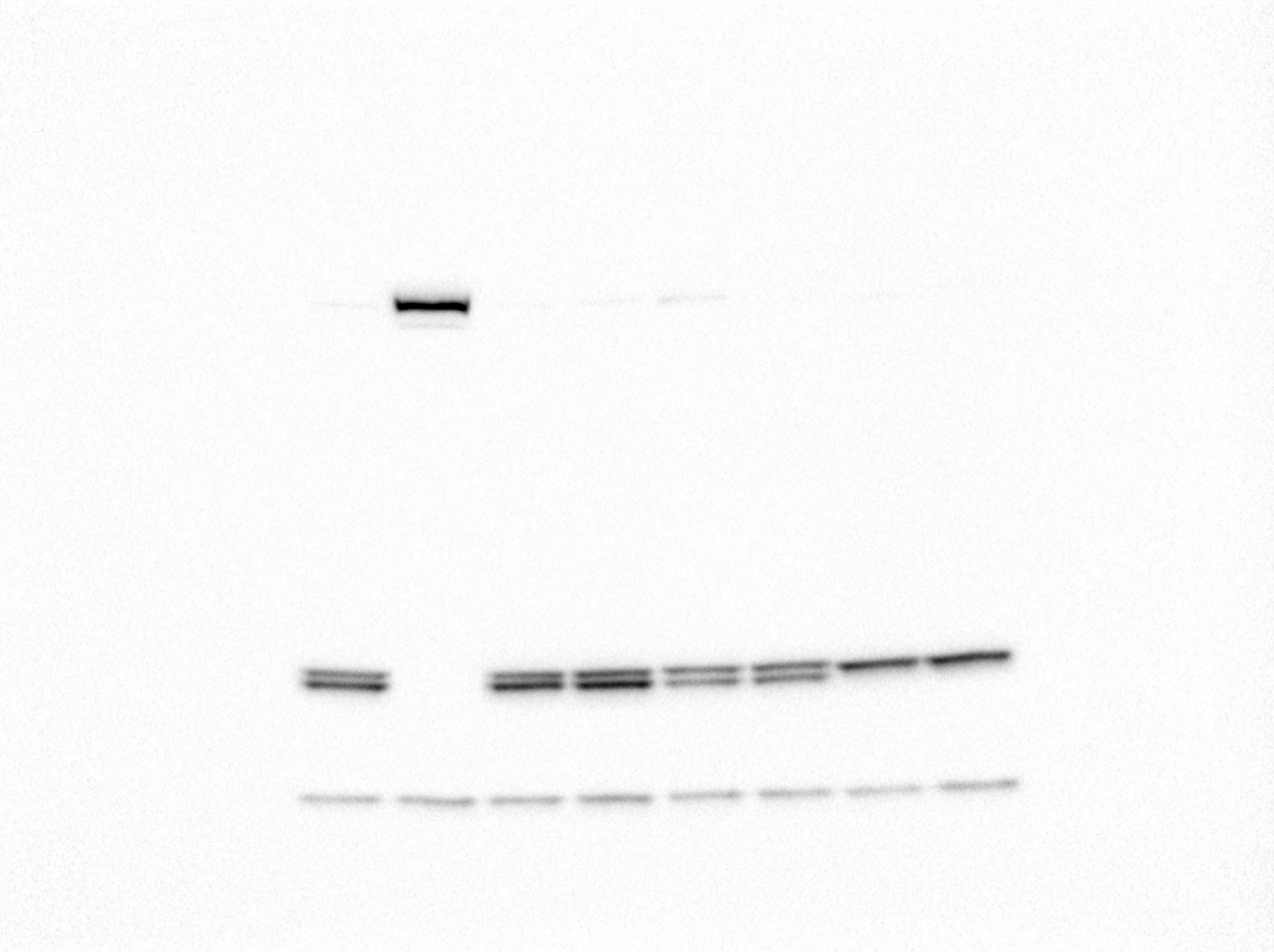

Supplement: Figure 5—figure supplement 1—source data 1. [file elife-81606-fig5-figsupp1-data1.zip › Figure 5-Figure supplement 1-source data /figure5-supplement1B_HA.jpg]

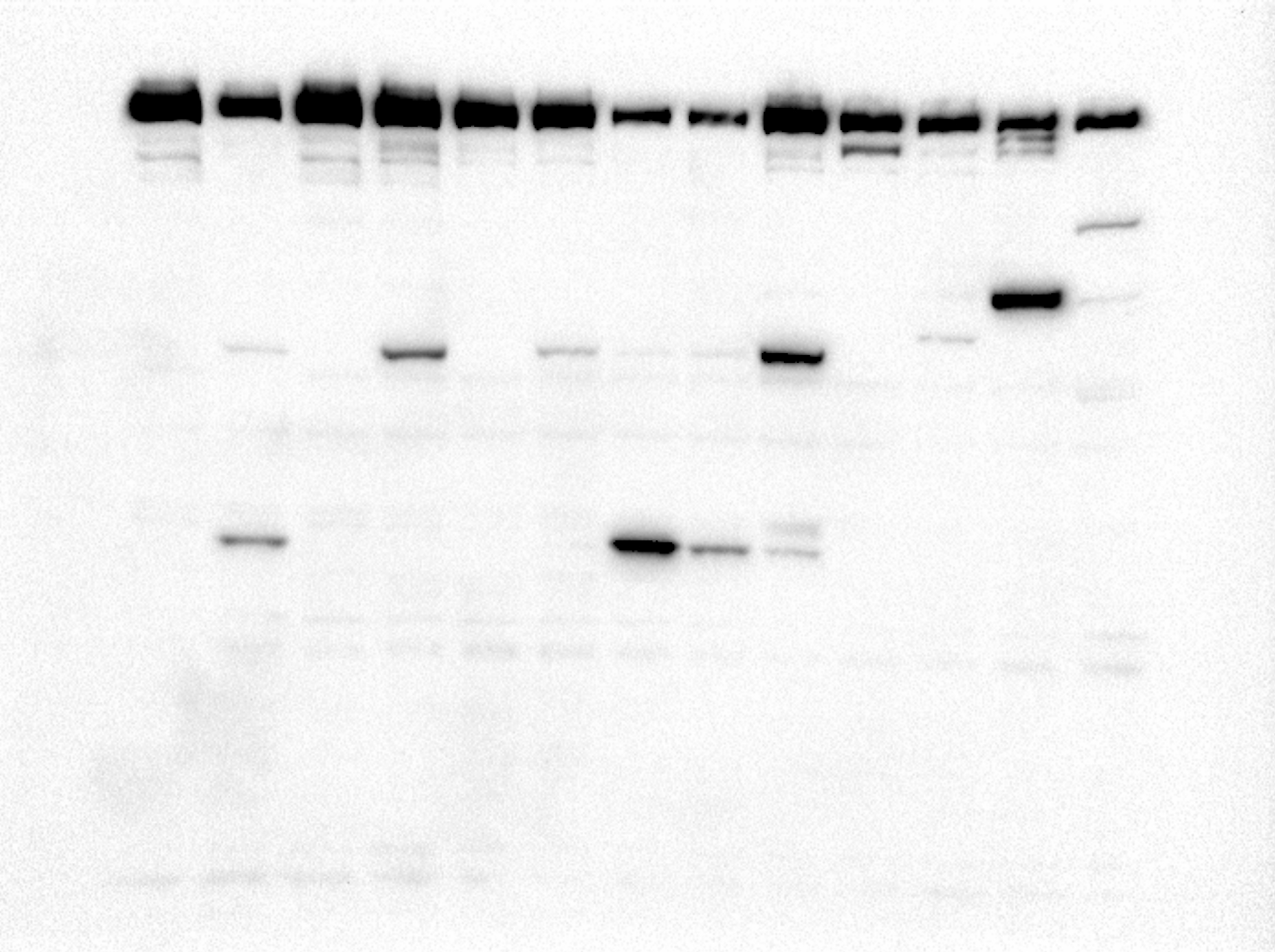

Supplement: Figure 5—figure supplement 1—source data 1. [file elife-81606-fig5-figsupp1-data1.zip › Figure 5-Figure supplement 1-source data /figure5-supplement1A_Myc.jpg]

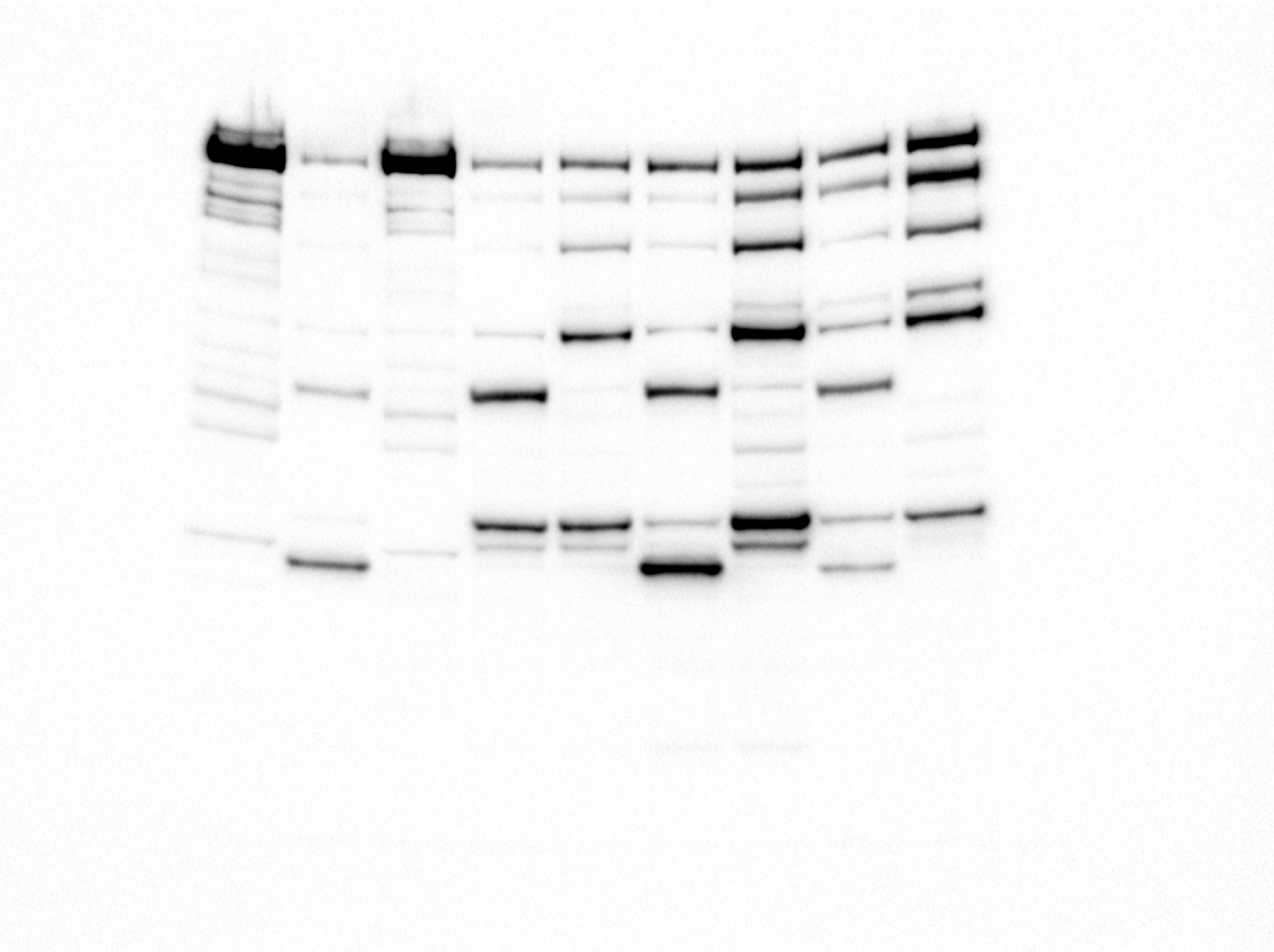

Supplement: Figure 5—figure supplement 1—source data 1. [file elife-81606-fig5-figsupp1-data1.zip › Figure 5-Figure supplement 1-source data /figure5-supplement1B_Myc.jpg]

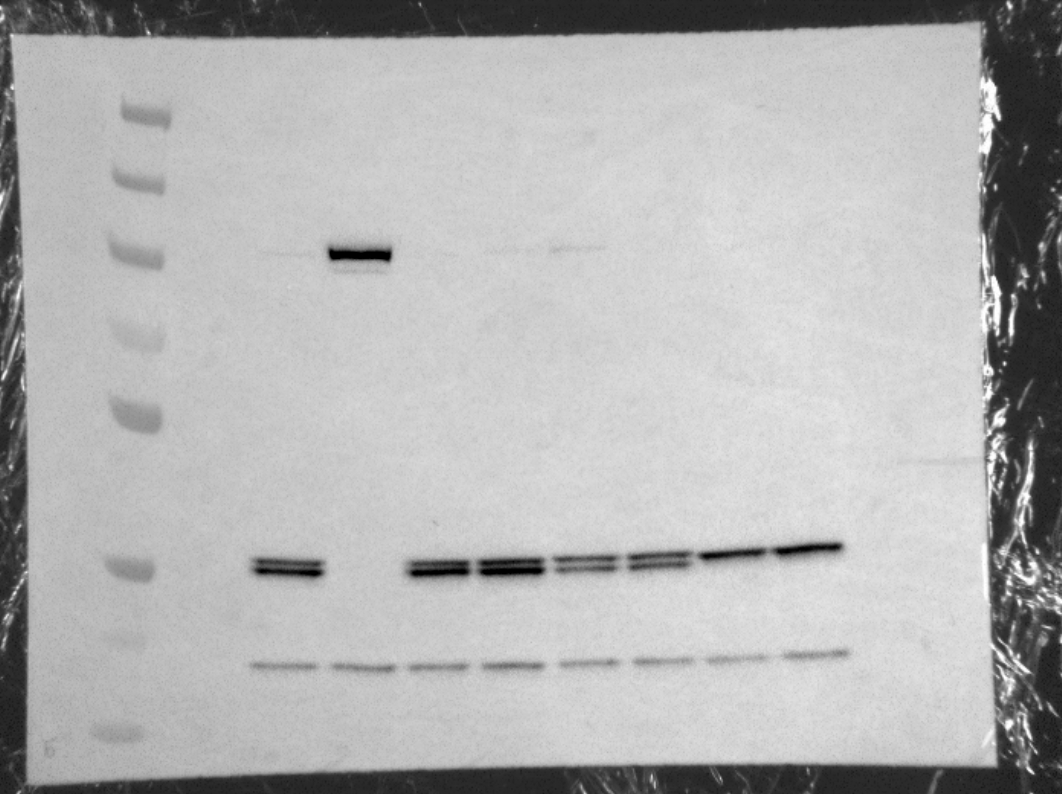

Supplement: Figure 5—figure supplement 1—source data 1. [file elife-81606-fig5-figsupp1-data1.zip › Figure 5-Figure supplement 1-source data /figure5-supplement1B_HA_merged.jpg]

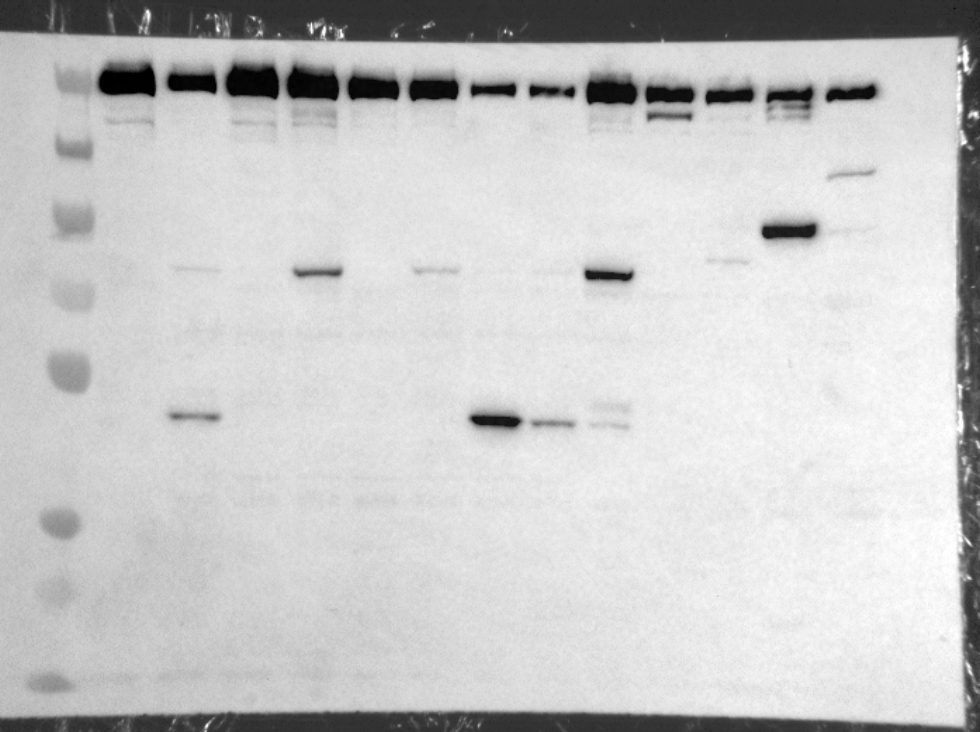

Supplement: Figure 5—figure supplement 1—source data 1. [file elife-81606-fig5-figsupp1-data1.zip › Figure 5-Figure supplement 1-source data /figure5-supplement1A_Myc_merged.jpg]

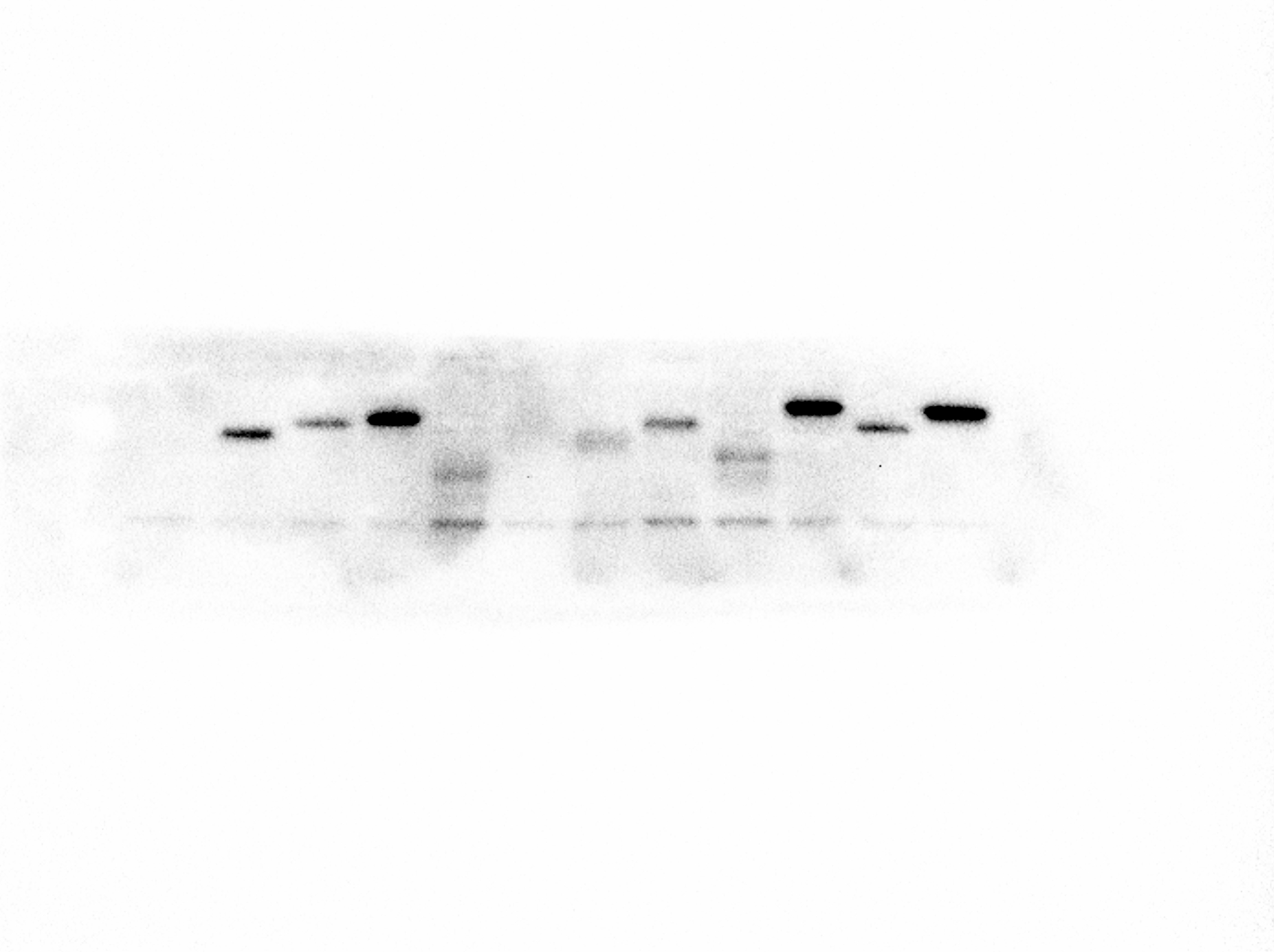

Supplement: Figure 5—figure supplement 1—source data 1. [file elife-81606-fig5-figsupp1-data1.zip › Figure 5-Figure supplement 1-source data /figure5-supplement1A_HA.jpg]

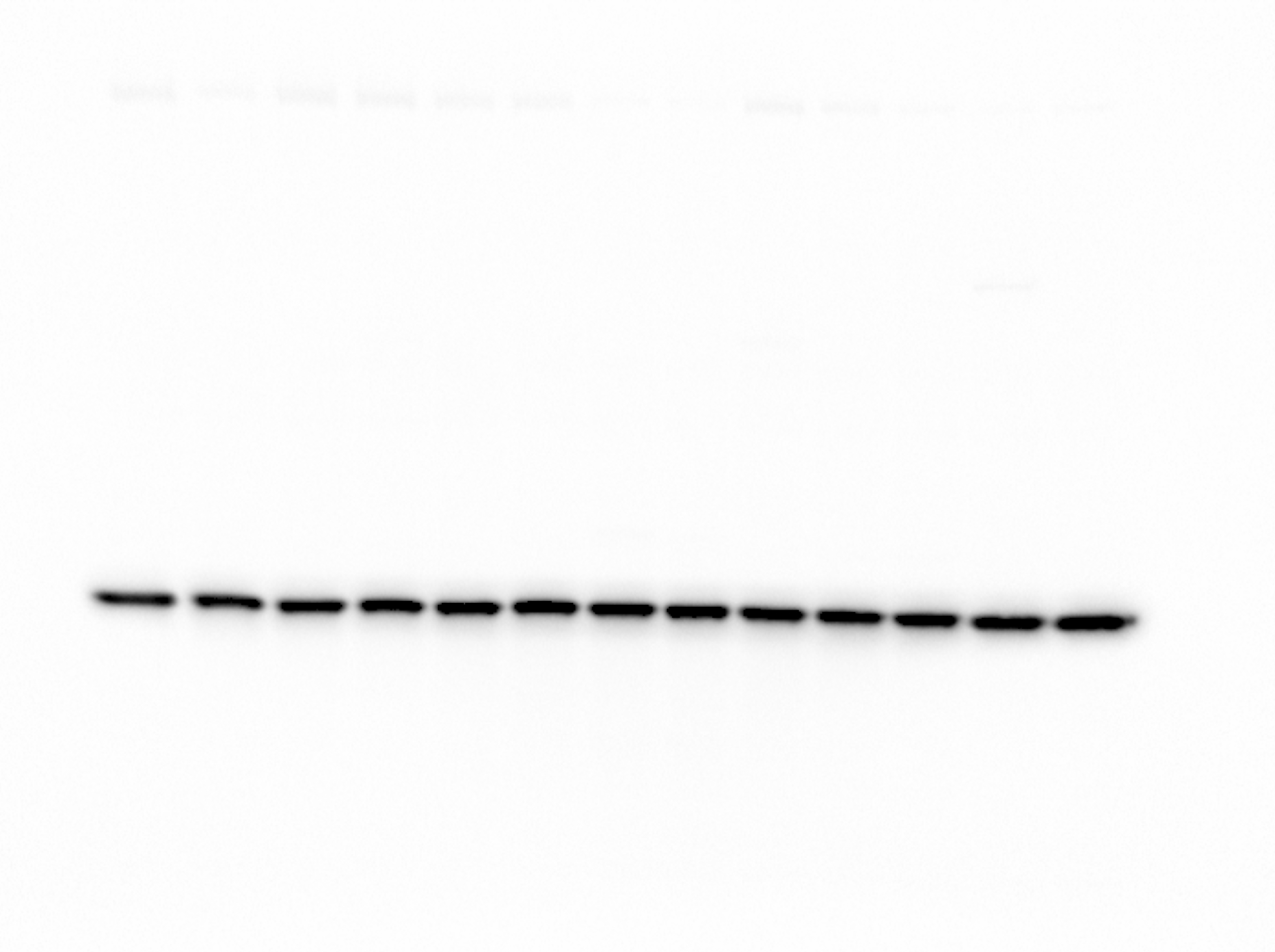

Supplement: Figure 5—figure supplement 1—source data 1. [file elife-81606-fig5-figsupp1-data1.zip › Figure 5-Figure supplement 1-source data /figure5-supplement1A_GAPDH.jpg]

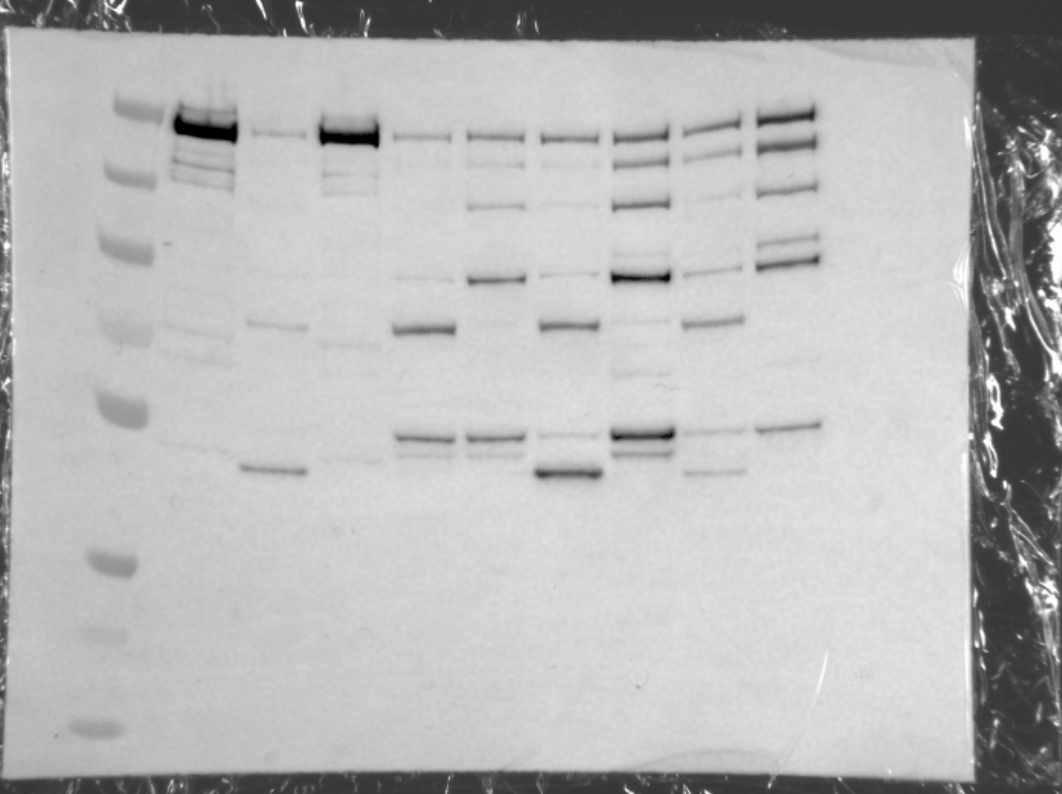

Supplement: Figure 5—figure supplement 1—source data 1. [file elife-81606-fig5-figsupp1-data1.zip › Figure 5-Figure supplement 1-source data /figure5-supplement1B_Myc_merged.jpg]

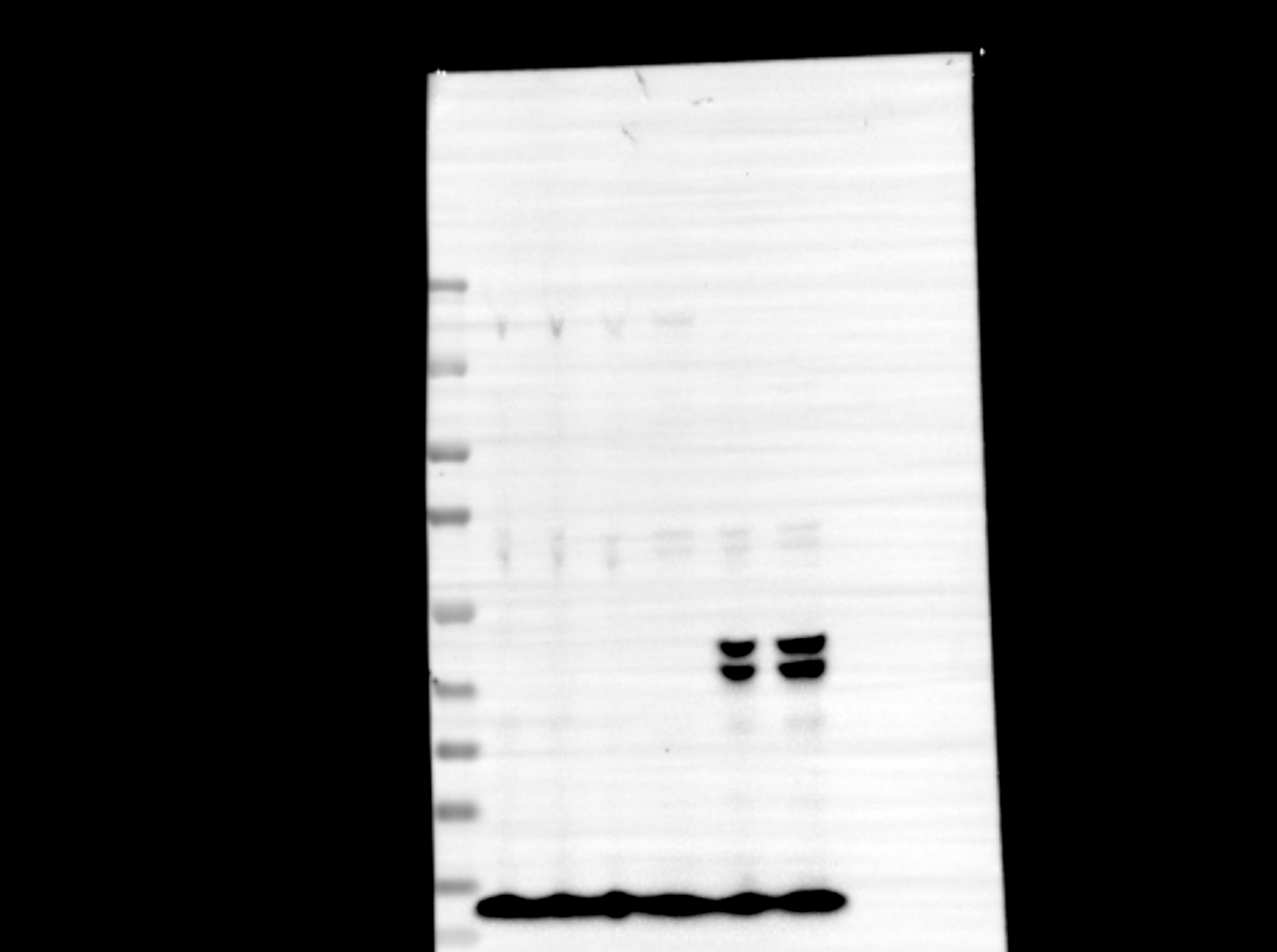

Supplement: Figure 6—source data 1. [file elife-81606-fig6-data1.zip › Figure 5/+3C Peroxisome Assay Cleavage_FKBP(Multichannel).tif]

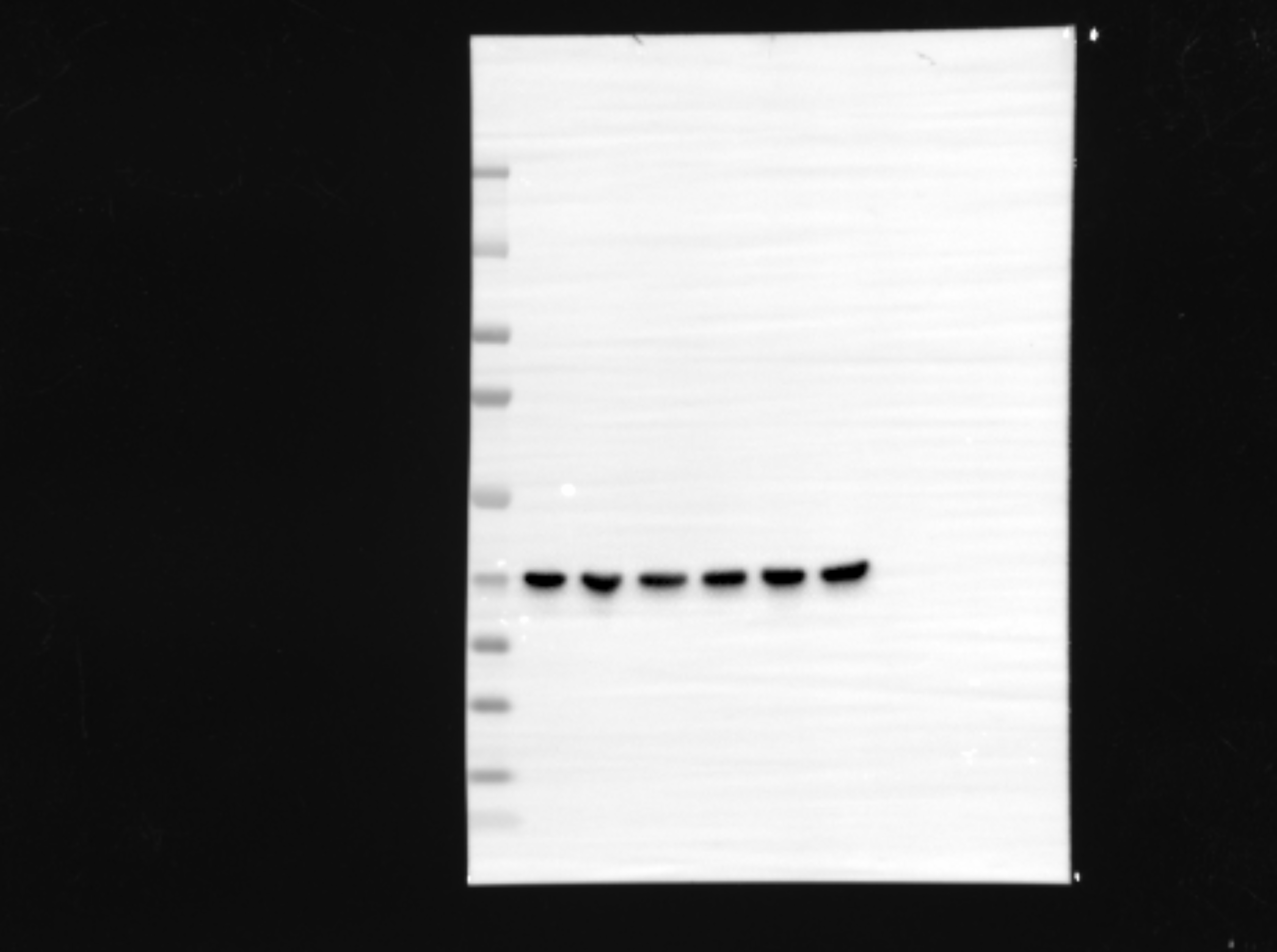

Supplement: Figure 6—source data 1. [file elife-81606-fig6-data1.zip › Figure 5/+3C Peroxisome Assay Cleavage_GAPDH(Multichannel).tif]

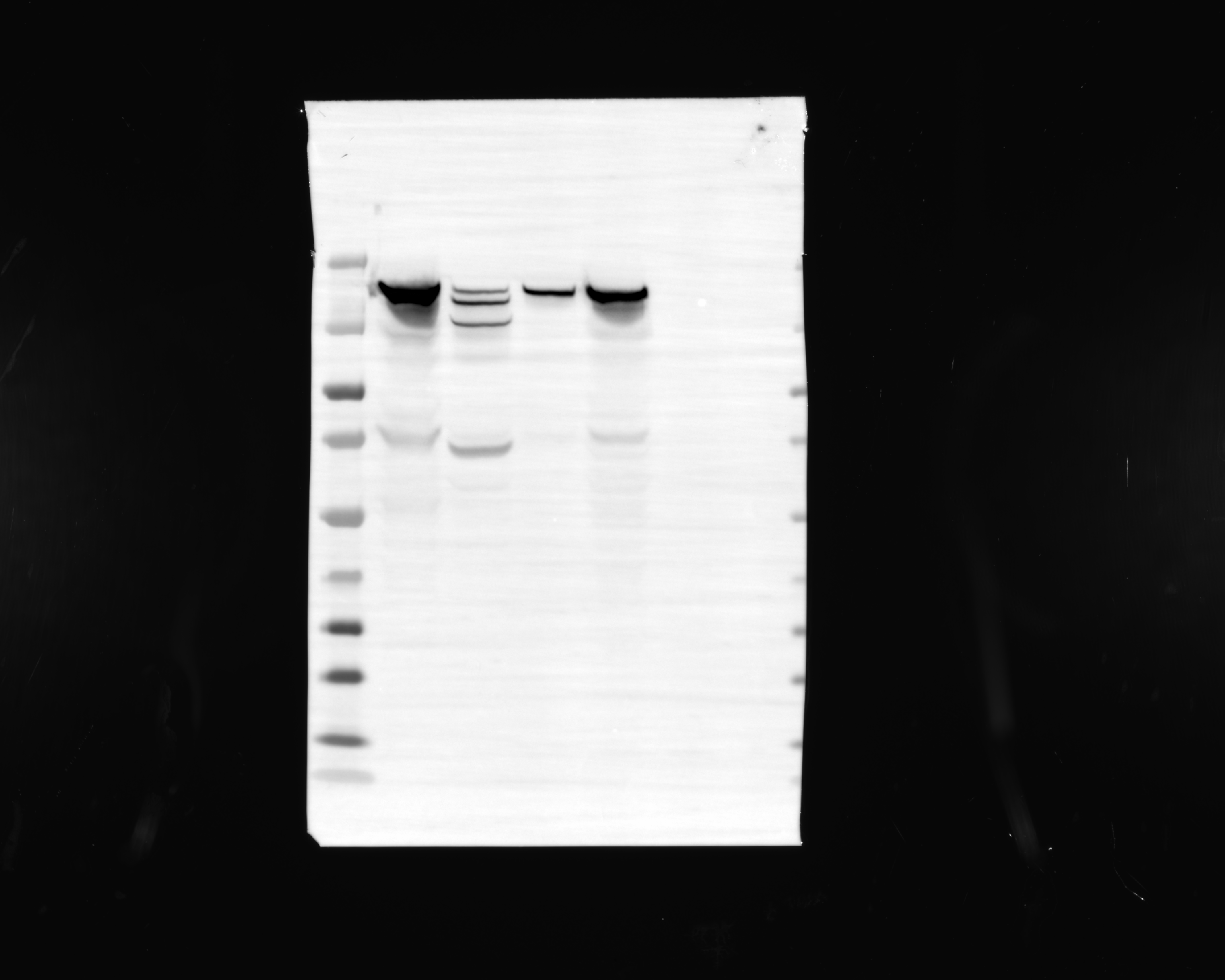

Supplement: Figure 6—source data 1. [file elife-81606-fig6-data1.zip › Figure 5/+3C Peroxisome Assay Cleavage_Flag(Multichannel).tif]
